# Burden of cancers in India - estimates of cancer crude incidence, YLLs, YLDs and DALYs for 2021 and 2025 based on National Cancer Registry Program

**DOI:** 10.1186/s12885-022-09578-1

**Published:** 2022-05-11

**Authors:** Vaitheeswaran Kulothungan, Krishnan Sathishkumar, Sravya Leburu, Thilagavathi Ramamoorthy, Santhappan Stephen, Dharmappa Basavarajappa, Nifty Tomy, Rohith Mohan, Geetha R. Menon, Prashant Mathur

**Affiliations:** 1 Indian Council Medical Research (ICMR) – National Centre for Disease Informatics and Research (NCDIR), Nirmal Bhawan-ICMR Complex (II Floor), Poojanahalli, Kannamangala Post, Bengaluru, Karnataka 562 110 India; 2 National Health Mission, Bengaluru, Karnataka India; 3 Indian Council Medical Research (ICMR) – National Institute of Medical Statistics (NIMS), New Delhi, India

**Keywords:** Burden, Cancer, Disability adjusted life years, Epidemiology, India

## Abstract

**Background:**

Cancer is the major cause of morbidity and mortality worldwide. The cancer burden varies within the regions of India posing great challenges in its prevention and control. The national burden assessment remains as a task which relies on statistical models in many developing countries, including India, due to cancer not being a notifiable disease. This study quantifies the cancer burden in India for 2016, adjusted mortality to incidence (AMI) ratio and projections for 2021 and 2025 from the National Cancer Registry Program (NCRP) and other publicly available data sources.

**Methods:**

Primary data on cancer incidence and mortality between 2012 and 2016 from 28 Population Based Cancer Registries (PBCRs), all-cause mortality from Sample Registration Systems (SRS) 2012–16, lifetables and disability weight from World Health Organization (WHO), the population from Census of India and cancer prevalence using the WHO-DisMod-II tool were used for this study. The AMI ratio was estimated using the Markov Chain Monte Carlo method from longitudinal NCRP-PBCR data (2001–16). The burden was quantified at national and sub-national levels as crude incidence, mortality, Years of Life Lost (YLLs), Years Lived with Disability (YLDs) and Disability Adjusted Life Years (DALYs). The projections for the years 2021 and 2025 were done by the negative binomial regression model using STATA.

**Results:**

The projected cancer burden in India for 2021 was 26.7 million DALYs_AMI_ and expected to increase to 29.8 million in 2025. The highest burden was in the north (2408 DALYs_AMI_ per 100,000) and northeastern (2177 DALYs_AMI_ per 100,000) regions of the country and higher among males. More than 40% of the total cancer burden was contributed by the seven leading cancer sites — lung (10.6%), breast (10.5%), oesophagus (5.8%), mouth (5.7%), stomach (5.2%), liver (4.6%), and cervix uteri (4.3%).

**Conclusions:**

This study demonstrates the use of reliable data sources and DisMod-II tools that adhere to the international standard for assessment of national and sub-national cancer burden. A wide heterogeneity in leading cancer sites was observed within India by age and sex. The results also highlight the need to focus on non-leading sites of cancer by age and sex. These findings can guide policymakers to plan focused approaches towards monitoring efforts on cancer prevention and control. The study simplifies the methodology used for arriving at the burden estimates and thus, encourages researchers across the world to take up similar assessments with the available data.

**Supplementary Information:**

The online version contains supplementary material available at 10.1186/s12885-022-09578-1.

## Background

Cancer ranks either first or second among the leading causes of death before the age of 70 years across 91 out of the 172 countries worldwide [1]. The GLOBOCAN 2018, reported 18.1 million new cancer cases and 9.6 million deaths globally [2]. By 2040, the cancer incidence and mortality are expected to rise to 29.5 million and 16.3 million, respectively [2]. New and challenging problems — rapid urbanization, population ageing, inactive and unhealthy lifestyles, indoor and outdoor air pollution, etc., are responsible for the emerging cancer burden across the globe, majorly impacting the middle-to-low socio-economic countries including India [3].

There have been previous attempts to estimate the cancer burden in different parts of India [3–10]. The Global Burden of Disease (GBD) 2016 study, attributed 8.3% of deaths and 5% of disability-adjusted life years (DALYs) to cancer alone [3]. The GLOBOCAN 2018, reported 1.1 million cancer cases and more than 0.7 million cancer deaths [11]. The Medical Certification of Cause of Death, 2018 reported cancer as the fifth leading cause of death amounting to 5.7% of all deaths in India [12]. The cancer burden has shown a steady increase with an estimated 0.8 million new cancer cases every year [11]. In 2040, nearly 2 million new cancer cases and more than 1 million deaths are estimated [11]. The heterogeneities in cancer epidemiology within India are well-documented [3–10]. The latest publication from the National Cancer Registry Program (NCRP), India points to the differences in cancer incidence rates. Aizawl district of Mizoram showed 7 times higher incidence rates of cancer in males and 4 times in females from that in Osmanabad and Beed in Maharashtra [6, 7].

The population-based cancer registries (PBCRs) are the only reliable, long period sources of data on the magnitude and patterns of cancer in the country [6, 7]. The data has been used in multiple studies, including the GBD study, to estimate the cancer burden for India [3, 5]. The Indian Council of Medical Research (ICMR) - National Centre for Disease Informatics and Research (NCDIR) - NCRP is a valuable data repository on cancer since its establishment in 1981 [6, 7]. To date, only constructed methods have been adopted to estimate the DALYs for cancer as a metric to quantify cancer burden in India. These studies have used econometric models that have inconsistent methodology and provide diverse results over different years. Moreover, all these results would also depend on availability of incidence and mortality data. In India due to limited availability of mortality data we have used the real-time data on cancer incidence and mortality from the PBCRs. Thus, we aimed to analyze and report cancer burden estimates for 2021 and 2025 in India at the national and sub-national level using the simplest methods that can be easily adapted and replicated. Also, these methods can be easily applied by other similar countries having inadequate representative data sets. Such attempts have been made in the past by ICMR to assess the cancer burden [8]. Additionally, the study aimed to derive adjusted mortality to incidence (AMI) ratio for India. In this paper, we present the methodology, results and discuss the implications of cancer burden in India using PBCR-NCRP data of 2012–16.

## Methods

### Overview

As a continued effort to the ICMR – Burden of Disease (BOD) study in 2004 [8], the BOD – Noncommunicable Disease (NCD) study was undertaken by ICMR - Ministry of Health and Family Welfare, Government of India collaboration to update the burden estimates for 2015. The cancer burden aspect of the study was tasked to the ICMR-NCDIR, a nodal institute for the NCRP [6, 7] to assess metrics of cancer burden in India. Incidence, mortality, prevalence, years of life lost (YLLs), years lived with disability (YLDs) and DALYs were the metrics derived. This analysis supersedes the 2015 update with new projections for 2021 and 2025 using 2012–16 NCRP data.

### Data sources

To meet the specific data requirements for estimating the national burden of cancer metrics and its future projections, we adhered to freely available standard data sources (Additional figure 1a) and adapted the recommended GBD-WHO-DisMod II tool [13].

Cancer-specific data on incidence and mortality by age and sex for the period 2012–16 was extracted from the 28 NCRP-PBCRs, which collect data on all ‘ICD - O3’ behaviour code cancers [6, 7]. For every registry, the pooled incidence rate for quinquennial age groups for both sexes, every cancer site (C00 – C96) were calculated as the number of new cases reported per year divided by the mid-year population of that year. The number of deaths for every cancer site was divided by the mid-year population to determine the pooled mortality rates. The PBCR reported mortality to incidence (RMI) ratio for males and females in each age group was estimated by dividing the number of deaths from cancer with the corresponding incidence estimates for the defined year from the cancer registry data [6, 7].

Considering the limitations of under reporting in the mortality systems in India, we searched for available evidence on the national MI ratio. We came across two national studies reporting an average of 35.0 and 75.4% as MI ratio for India [4, 14]. Accordingly, longitudinal data points with reported MI ratio of ≥35.0% [14] from NCRP-PBCRs between 2001 and 2016 was extracted to derive at the standard MI ratio for India by sex. Total of 256 data points had an MI ratio of ≥35.0%, of these 150 were for males and 106 for females. The Akaike’s and Bayesian Information criterion showed that the Gamma distribution as the best fit to the data *(*R software*, version 4.1.2 R Core Team, Vienna, Austria)* (Additional figure 1b). The AMI was derived from the Markov Chain Monte Carlo (MCMC) method *(*STATA 14.2*, StataCorp, College Station, Texas, USA)*. The estimated mean of AMI converged after 10,000 iterations was found to be 50.3% (95% uncertainty interval (UI): 48.7–51.9) for males and 46.6% (95% Ul: 44.9–48.4%) for females. We replaced this mean AMI for those registries (2012–16) with RMI < 50.3% (males) and < 46.6% (females). The results thus derived from the AMI are presented as YLL_AMI_, YLD_AMI_ and DALY_AMI_ for all cancer by sex.

The state-level population was obtained from the 2001 and 2011 Census of India, to arrive at the projected population by age and sex from 2012 to 2016 using the difference distribution method [15]. Age and sex-specific all-cause mortality were procured from the Sample Registration System (SRS), Office of Registrar General of India for 2012–16 [16]. The standard lifetables provided by WHO were used to arrive at the life expectancy for age groups and sex [17] (Additional Tables 1, 2 and 3). The disability weight (DW) for cancer were obtained from the published GBD–2004 update by WHO. The DW for malignant neoplasm and their long-term sequel is 0.75 for all the metastatic stages of cancer [18]. The prevalence of cancer by type, age and sex were obtained using the cancer incidence, mortality, MI ratio, population and all-cause mortality rate as inputs in the DisMod-II tool [5, 13] (Additional Figure 1a).

The 28 states and 2 out of 8 Union Territories (Delhi and Jammu & Kashmir) were grouped into six regions based on the pooled NCRP-PBCR reporting for regions [6]. The regions with existing PBCRs were the data sources for those respective regions corresponding to their location and, regions that had less number or lacked PBCRs, the nearest PBCRs data was used (Additional Table 4a) [6].

### Statistical analyses

To obtain YLLs, the total number of deaths due to cancer in a defined age group was multiplied by the standard life expectancy of that age group. YLD estimates were generated by multiplying the total number of prevalent cancer cases at respective age groups by disability-weight of cancer. DALY metrics were the sum of the YLL and YLD estimates [5]. All cancer burden metrics — YLL, YLD and DALY as well as YLL_AMI_, YLD_AMI_ and DALY_AMI_ were estimated in age-standardized rates (ASR) using the WHO World Population Standard distribution (2000–2025) to assess the comparability of results with previous GBD estimates [19].

The cancer burden metrics projections for 2021 and 2025 (using adjusted MI ratio) was done assuming the pooled incidence and mortality rates obtained from 28 PBCRs that represents India’s incidence and mortality from cancer and age-specific cancer incidence. With the obtained information on cancer type, sex and age groups, the cancer burden estimates at the national and sub-national level were projected using 16 years of data between 2001 and 16 [20]. The negative binomial regression model was applied using STATA 14.2 (StataCorp, College Station, Texas, USA) to project the cancer burden metrics for 2021 and 2025. This model was preferred over the Poisson regression model as the conditional variance of the cancer burden metrics were greater than the conditional mean. We applied the Lagrange multiplier test to examine the presence of over dispersion in the data. The results for all cancers are presented as YLL, YLD, DALY with reported MI ratio and YLL_AMI_, YLD_AMI_, DALY_AMI_ with adjusted MI ratio per 100,000 population.

## Results

### National and sub-national burden of cancer in India

**Fig. 1:**
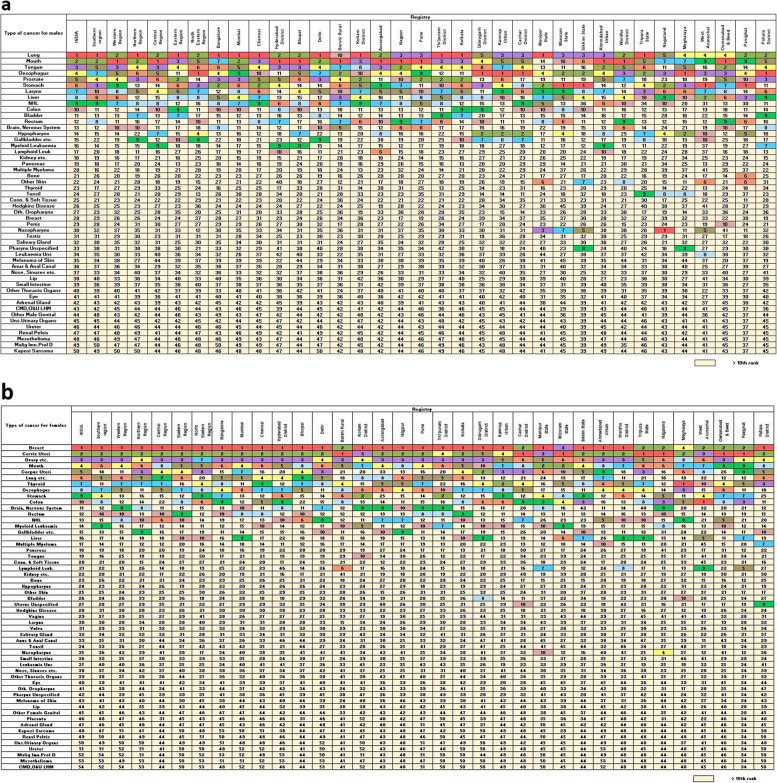
**a** Ranking of crude incidence rate of leading cancer sites in males for India, by region and PBCR. **b** Ranking of crude incidence rate of leading cancer sites in females for India, by region and PBCR

In 2016, men from southern, northern and eastern regions had the highest crude incidence rates of lung cancer, while mouth and oesophageal cancers ranked first in the western, central and northeast regions of the country followed by lung cancer. Oesophageal cancer ranked fifth to eleventh in incidence in other regions (Fig. 1a). In females, breast cancer and cervical cancer ranked first and second in incidence irrespective of regions, while ovarian cancer occupied the third rank across all regions except in the north and northeast parts of India. Mouth cancer ranked fourth to eighth and oesophageal cancer was between fifth – eleventh rank in incidence (Fig. 1b). Registry wise information has been provided in Additional Table 5.

**Fig. 2:**
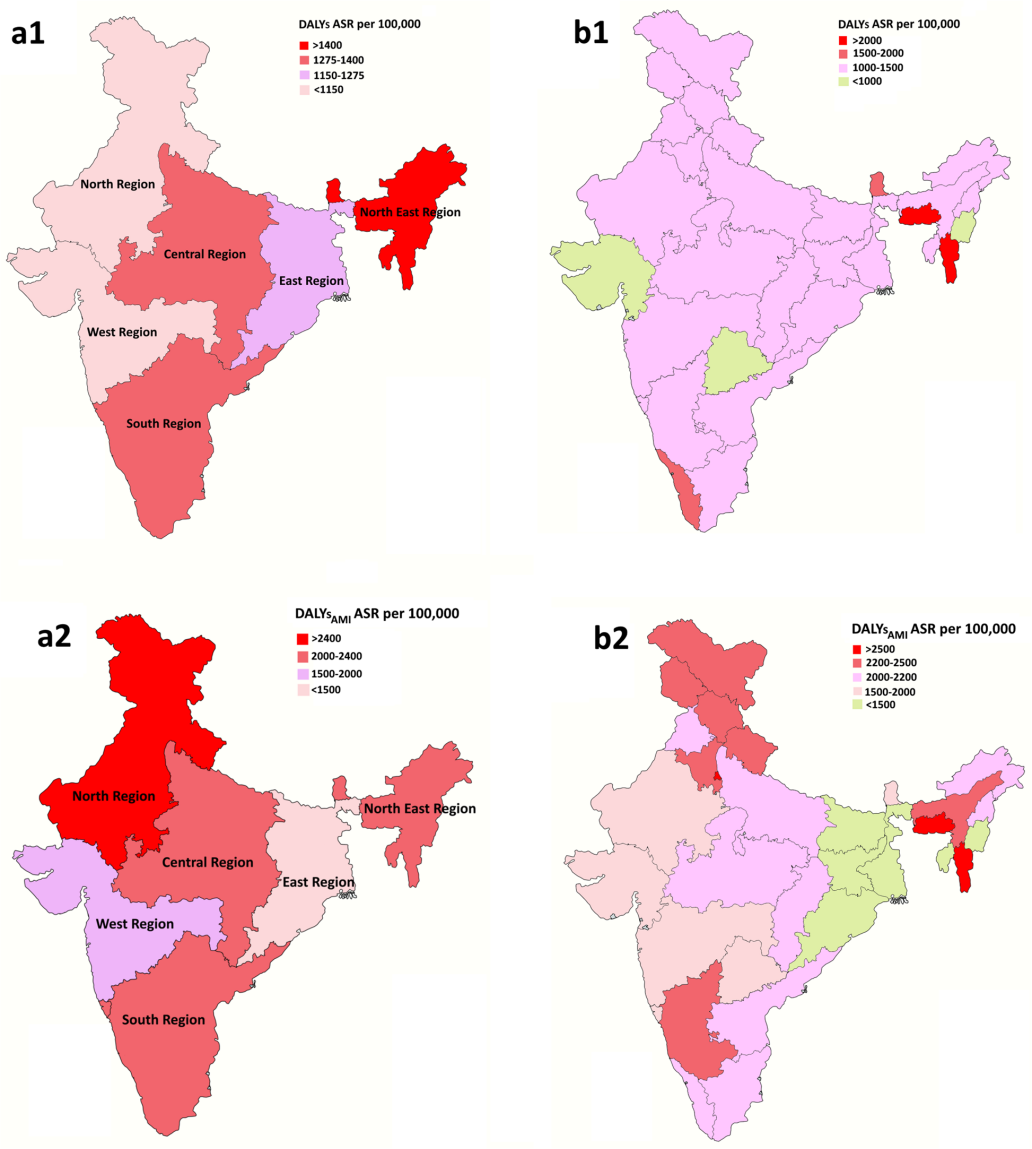
Distribution of total cancer DALYs – ASR per 100,000 by (**a**) Region and (**b**) State

The total estimated burden of cancer for India in 2016 was 1277 DALYs per 100,000. After adjusting the MI ratio, the national cancer burden was 1908 DALYs_AMI_ per 100,000 (Additional Table 4a). The burden imposed from cancer for males was higher than females (Additional Table 4b and c). The majority of the cancer burden was in the northeast region (1428 DALYs per 100,000), followed by southern (1353 DALYs per 100,000) and central (1351 DALYs per 100,000) regions of India. After adjusting the MI ratio, north (2408 DALYs_AMI_ per 100,000), north-east (2177 DALYs_AMI_ per 100,000) southern (2138 DALYs_AMI_ per 100,000) and central (2024 DALYs_AMI_ per 100,000) regions had the highest DALYs_AMI_ from cancer. North-east and northern India (103 YLDs_AMI_ per 100,000) showed the highest YLDs_AMI_ (Additional Table 4a and Fig. 2). Across all the states/UTs, Mizoram (3424 DALYs_AMI_ per 100,000) followed by Delhi (2651 DALYs_AMI_ per 100,000) and Meghalaya (2609 DALYs_AMI_ per 100,000) had the highest cancer DALYs. Mizoram (153 per 100,000) and Arunachal Pradesh (140 per 100,000) had the highest YLDs_AMI_ from cancer (Additional Table 4a).

### Cancer burden by site and sex

The top five leading causes of cancer DALYs in 2016 for males were lung (183.3 per 100,000), mouth (89.6 per 100,000), oesophagus (89.4 per 100,000), stomach (76.7 per 100,000) and liver cancers (74.9 per 100,000) excluding the other and unspecified sites. Prostate cancer contributed to 6.5 YLDs per 100,000 third to lung and mouth cancers. The most common causes of cancer DALYs among females were breast (232.7 per 100,000), cervix uteri (98.6 per 100,000), ovary (78.9 per 100,000), lung (74.1 per 100,000) and gall bladder (58.3 per 100,000) cancers (Table 1), (Additional Table 6).

**Table 1:** Site-specific burden of cancer (YLLs, YLDs, DALYs) per 100,000 by sex in 2016

ICD-10	Cancer site	YLLs-ASR			YLDs-ASR			DALYs-ASR			% YLLs to DALYs
		Males	Females	Total	Males	Females	Total	Males	Females	Total	
C00	Lip	3.5	1.7	2.6	0.5	0.2	0.3	3.9	1.9	2.9	88.3
C01-C02	Tongue	61.4	20.8	41.1	5.7	2.0	3.9	67.2	22.8	45.0	91.4
C03-C06	Mouth	81.6	34.1	57.8	8.0	3.4	5.7	89.6	37.4	63.5	91.0
C07-C08	Salivary Gland	3.7	2.0	2.9	0.8	0.7	0.8	4.5	2.7	3.6	79.0
C09	Tonsil	11.8	2.1	6.9	1.1	0.3	0.7	13.0	2.3	7.7	90.7
C10	Other Oropharynx	12.4	2.1	7.2	0.9	0.2	0.6	13.3	2.3	7.8	92.6
C11	Nasopharynx	7.9	3.3	5.6	0.7	0.4	0.5	8.6	3.6	6.1	91.7
C12-C13	Hypopharynx	29.8	7.9	18.8	2.7	0.6	1.7	32.5	8.5	20.5	91.9
C14	Pharynx Unspecified	11.3	3.5	7.4	0.5	0.2	0.3	11.8	3.6	7.7	95.5
C15	Oesophagus	84.3	44.3	64.3	5.1	3.1	4.1	89.4	47.4	68.4	94.0
C16	Stomach	72.3	41.9	57.1	4.4	2.4	3.4	76.7	44.3	60.5	94.4
C17	Small Intestine	3.6	2.6	3.1	0.3	0.2	0.3	3.9	2.9	3.4	91.9
C18	Colon	31.1	25.6	28.4	2.9	2.2	2.5	34.0	27.8	30.9	91.8
C19-C20	Rectum	29.8	21.4	25.6	2.8	1.9	2.4	32.6	23.4	28.0	91.6
C21	Anus and Anal cavity	3.9	2.9	3.4	0.5	0.3	0.4	4.3	3.2	3.8	89.3
C22	Liver	71.5	32.6	52.1	3.4	1.5	2.4	74.9	34.1	54.5	95.6
C23-C24	Gallbladder	29.4	54.7	42.1	2.1	3.6	2.8	31.5	58.3	44.9	93.7
C25	Pancreas	29.4	20.6	25.0	1.6	1.1	1.3	31.0	21.7	26.4	95.0
C30-C31	Nose, Sinuses	4.0	1.9	3.0	0.5	0.4	0.4	4.4	2.3	3.4	87.7
C32	Larynx	38.8	5.6	22.2	4.2	0.6	2.4	43.0	6.2	24.6	90.2
C33-C34	Lung	173.5	70.3	121.9	9.8	3.8	6.8	183.3	74.1	128.7	94.7
C37-C38	Other Thoracic Organs	4.0	2.7	3.3	0.3	0.2	0.2	4.2	2.8	3.5	93.7
C40-C41	Bone	13.8	10.2	12.0	1.1	0.8	1.0	14.9	11.0	12.9	92.6
C43	Melanoma of Skin	2.3	1.7	2.0	0.5	0.5	0.5	2.8	2.2	2.5	80.9
C44	Other Skin	9.1	6.9	8.0	1.3	1.3	1.3	10.5	8.2	9.3	86.0
C45	Mesothelioma	0.2	0.1	0.2	0.1	0.1	0.1	0.3	0.2	0.2	63.6
C46	Kaposi Sarcoma	0.2	0.3	0.2	0.0	0.2	0.1	0.2	0.5	0.3	62.0
C47 + C49	Connective & Soft Tissue	8.8	7.3	8.0	1.1	1.0	1.1	10.0	8.2	9.1	88.4
C50	Breast	5.7	203.6	104.6	0.8	29.1	15.0	6.5	232.7	119.6	87.5
C51	Vulva	0.0	2.4	1.2	0.0	0.4	0.2	0.0	2.8	1.4	85.0
C52	Vagina	0.0	3.2	1.6	0.0	0.5	0.3	0.0	3.7	1.9	86.2
C53	Cervix Uteri	0.0	87.6	43.8	0.0	11.0	5.5	0.0	98.6	49.3	88.9
C54	Corpus Uteri	0.0	14.9	7.5	0.0	3.9	1.9	0.0	18.8	9.4	79.4
C55	Uterus Unspecified	0.0	7.8	3.9	0.0	0.6	0.3	0.0	8.4	4.2	93.4
C56	Ovary	0.0	72.9	36.5	0.0	5.9	3.0	0.0	78.9	39.4	92.5
C57	Other Female Genital organs	0.0	0.6	0.3	0.0	0.1	0.1	0.0	0.7	0.4	84.1
C58	Placenta	0.0	0.3	0.2	0.0	0.7	0.4	0.0	1.1	0.5	31.0
C60	Penis	5.5	0.0	2.7	0.9	0.0	0.4	6.4	0.0	3.2	86.0
C61	Prostate	40.3	0.0	20.2	6.5	0.0	3.3	46.9	0.0	23.4	86.0
C62	Testis	4.5	0.0	2.3	0.6	0.0	0.3	5.1	0.0	2.6	88.7
C63	Other Male Genital organs	0.4	0.0	0.2	0.2	0.0	0.1	0.6	0.0	0.3	66.5
C64	Kidney	16.6	7.3	12.0	1.8	0.8	1.3	18.4	8.1	13.3	90.1
C65	Renal Pelvis	0.2	0.1	0.1	0.1	0.2	0.1	0.3	0.3	0.3	50.8
C66	Ureter	0.2	0.1	0.2	0.1	0.1	0.1	0.3	0.2	0.3	63.0
C67	Bladder	21.3	6.0	13.6	3.5	0.9	2.2	24.8	6.9	15.8	86.1
C68	Unspecified Urinary Organs	0.7	0.2	0.5	0.1	0.1	0.1	0.8	0.3	0.6	84.4
C69	Eye	1.5	0.8	1.2	0.4	0.5	0.4	1.9	1.3	1.6	72.8
C70-C72	Brain, Nervous System	37.7	26.1	31.9	2.2	1.5	1.9	39.9	27.6	33.7	94.5
C73	Thyroid	5.2	9.3	7.2	4.1	3.7	3.9	9.3	13.0	11.1	65.1
C74	Adrenal Gland	1.2	1.0	1.1	0.4	0.2	0.3	1.7	1.2	1.4	77.6
C81	Hodgkin’s Disease	7.5	4.5	6.0	1.0	0.6	0.8	8.5	5.1	6.8	88.6
C82-C85	Non-Hodgkin's Lymphoma	38.9	24.1	31.5	2.9	1.9	2.4	41.8	26.0	33.9	92.9
C88	Malignant Immunoproliferative Diseases	0.3	0.3	0.3	0.04	0.00	0.0	0.3	0.3	0.3	93.7
C90	Multiple Myeloma	15.5	12.8	14.1	1.4	1.1	1.2	16.9	13.8	15.4	92.0
C91	Lymphoid Leukaemia.	30.8	20.7	25.7	1.8	0.9	1.3	32.5	21.6	27.1	95.0
C92-C94	Myeloid Leukaemia	38.8	31.6	35.2	1.8	1.3	1.5	40.6	32.9	36.7	95.9
C95	Leukaemia Unspecified	14.7	10.8	12.8	0.4	0.4	0.4	15.1	11.2	13.2	97.0
C96	CMD, O&U LHM	1.3	0.8	1.0	0.1	0.1	0.1	1.4	0.9	1.1	91.3
O&U^a^	Other and unspecified	141.6	113.6	127.6	6.0	4.3	5.1	147.6	117.8	132.7	96.1
**All cancer sites**		**1268.5**	**1097.4**	**1183.0**	**91.8**	**98.3**	**95.1**	**1360.39**	**1195.8**	**1278.1**	**92.6**

^a^O&U includes the Sites (ICD-10:C26, C39, C48, C75, C76, C77, C78, C79, C80, C97)

*CMD* Chronic Myeloproliferative Disease, *O&U LHM* Other and unspecified malignant neoplasms of lymphoid, hematopoietic and related tissue

**Fig. 3:**
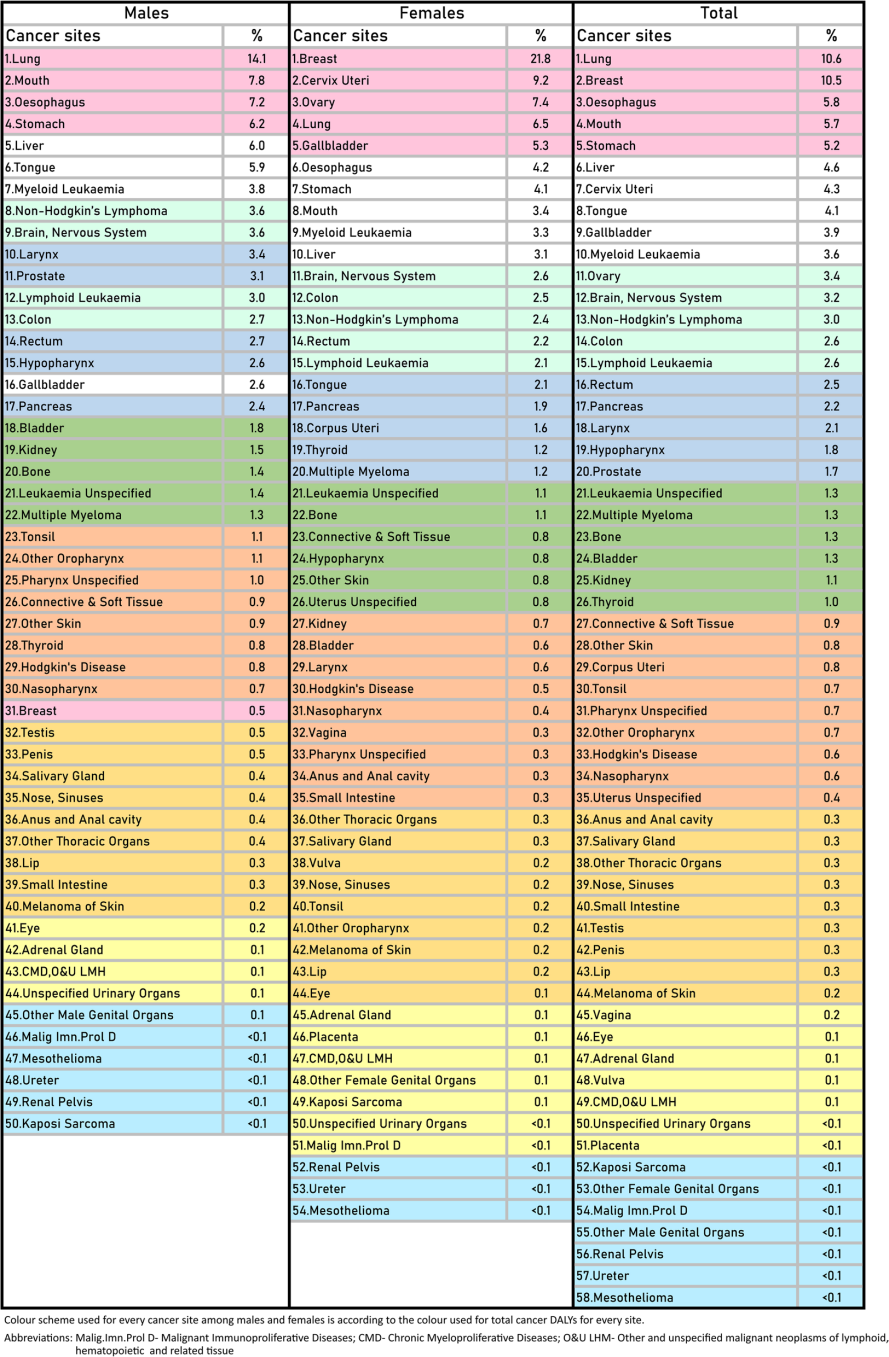
Contribution of DALYs from cancer by different sites, sex and overall (Percentage)

More than 5% of total cancer DALYs combined for both sexes occurred from the top five leading cancers — lung (10.6%), breast (10.5%), oesophageal (5.8%), mouth (5.7%), stomach (5.2%) cancers in 2016. The five leading causes of cancer DALYs for males were lung (14.1%), mouth (7.8%), oesophageal (7.2%), stomach (6.2%) and liver (6.0%) cancers that contributed to more than 6% of total DALYs. For females, the top five causes of more than 5% DALYs were breast cancer (21.8%), cervical cancer (9.2%), ovarian cancer (7.4%), lung cancer (6.5%) and gall bladder cancer (5.3%) (Fig. 3).

### Proportion contribution of cancer burden (YLLs, YLDs and DALYs) among five-year age groups

Cancer burden in 2016, showed a steep two-fold rise in age-standardized YLLs after 45–69 years, with highest at 65–69 years (14.0%) and 75–79 years (13.6%). Thereafter, there was a decrease with advancing age. The YLLs in males were highest at 75–79 years (14.9%) than at 65–69 years (14.4%), while in females they peaked at 65–69 years (14.0%) (Additional Figure 2a).

Both sexes combined, the proportion of YLDs increased with age, and subsequently, after 45–49 years there was a nearly four-fold increase in YLDs. The highest YLDs was observed between 75 and 79 years (14.6%) and decreased thereafter. Among females, YLDs were highest in the age group of 70–74 years (13.1%) (Additional Figure 2b).

With advanced age, the cancer DALYs increased from 30 to 34 years up to 75–79 years and subsequently decreased. The highest DALYs were in 65–69 years (13.9%) and at 85+ years (4.3%) the proportion of DALYs was nearly similar to burden at 40–44 years (3.5%). The DALYs were highest at 75–79 years (15.0%) in males and 65–69 years (13.9%) in females (Additional Figure 2c).

### Distribution of total cancer DALYs in percentage (%) by age group and site

Among both sexes, the highest DALYs at 0–14 years were from cancer of eye (50.7%), lymphoid leukaemia (44.6%); and at 15–34 years it was from cancer of testis (53.1%) and malignant bone tumours (37.9%). Nearly 24 cancer sites added to more than 50% of total cancer DALYs at 35–59 years, the highest from cancer of cervix uteri and breast (63.5% each). While at 60+ years, cancer of prostate and ureter contributed to 83.2% and 73.0% cancer DALYs, respectively. Thus, cancer prostate and ureter contributed to the highest DALYs amongst all other cancer sites irrespective of age (Additional Figure 3).

The leading site of lung contributed to the highest percentage of DALYs in the age group 60+ (51.5%) followed by 35–59 years (45.1%). While cancer breast showed the highest DALYs in percentage among those aged between 35 and 59 years (63.5%), followed by 60+ years (28.6%). Cancer oesophagus (54.9%), mouth (61.3%) and stomach (51.8%) contribute to the highest cancer DALYs between 35 and 59 years (Additional Figure 3).

### Projections for 2021 and 2025

In India, the burden for cancer was projected to be 26.7 million DALYs_AMI_ in 2021 and 29.8 million DALYs_AMI_ in 2025. The burden was higher among males than females (Table 2). Males contributed to 14.7 million YLLs_AMI_, 0.72 million YLDs_AMI_ and 15.5 DALYs_AMI_, whereas females contributed to 13.6 million YLLs_AMI_, 0.69 million YLDs_AMI_ and 14.3 DALYs_AMI_ from cancer in 2025 (Table 2).

**Table 2:** Projection estimates of YLLs_AMI_, YLDs_AMI_ and DALYs_AMI_ for cancer by sex for 2021 and 2025

Cancer burden	Males		Females		Total	
	2021	2025	2021	2025	2021	2025
**YLLs**_**AMI**_	13,022,317	14,732,919	12,459,724	13,614,452	25,496,645	28,368,793
**YLDs**_**AMI**_	620,767	722,140	615,286	693,876	1,236,981	1,417,760
**DALYs**_**AMI**_	13,640,795	15,450,483	13,072,149	14,302,783	26,728,484	29,776,432

### Change in cancer DALYs per 100,000 from 2004 to 2021

**Fig. 4:**
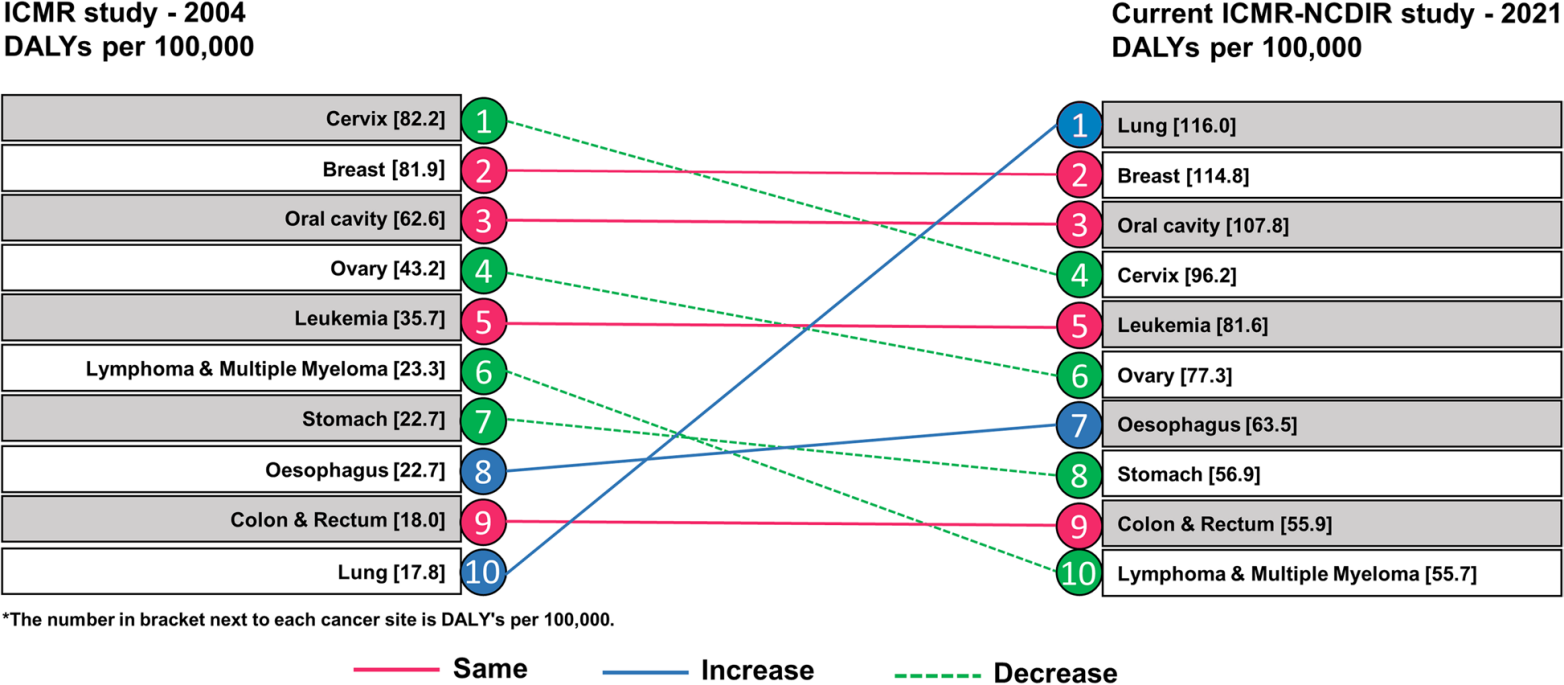
Change in DALYs per 100,000 by cancer site from 2004 to 2021 in India

When examining the DALYs by cancer sites from 2004 to 2021, cancer lung showed an increase in DALYs from tenth place to first. The number of DALYs contributed by oesophageal cancer was expected to increase from 22.7 per 100,000 to 63.5 per 100,000 moving from eighth to seventh place in 2021. Cancer cervix, ovary, lymphoma and multiple myeloma and stomach that led rankings in 2004 showed decline in 2021 (Fig. 4).

## Discussion

As per the National Health Policy 2017 of India, the estimation of DALYs is recommended as a key epidemiological tool to assess epidemiological transitions and study macro-level policies on the expected health care use, evaluate the impact of prevention and control programs, allocate resources, and benchmark the progress being made in the country [21–24]. This study provides robust country-specific burden imposed by cancer using the PBCR data (2012–16). However, challenges arise in providing timely data updates on cancer burden to the government due to existing limitations in the cancer registration and reporting systems. Real time availability of cancer incidence is lacking by 2–3 years and underreporting of deaths with inaccuracy in reporting of cancer specific deaths in the Civil Registration System would have its effects on estimation of AMI ratio. In addition, PBCRs cover approximately 10% of total population and the distribution of PBCR location and coverage is not uniform across all the states. Nevertheless, the burden imposed by cancer reinforces the need to view cancer burden metrics with the available data sources in the best possible context to inform cancer care and prevention strategies. This study calculated the burden of cancer in a standardized way in a developing country with limited information, and must be read with an understanding of the existing limitations in the reporting systems.

The main strengths of this study include the use of original, reliable and robust data to estimate national, regional and state-level cancer burden in India. Primarily, we used the ICMR-NCDIR-NCRP data as a source on cancer incidence and mortality in India and other established national data sources locally available to India — Census of India and SRS [6, 12, 13]. The use of WHO-DisMod II enables the replication of such studies by other researchers as well [5, 8, 25]. With better coverage of the PBCRs, quality and access to data in recent times, quantifying cancer burden from available data resources has improved [7]. Additionally, the study estimated standardized MI ratio derived from real-time longitudinal PBCR data points. The combination of sources and methods used in this study to estimate cancer burden are easily reproducible by other investigators.

There was an evident increase in the reported proportion of deaths from neoplasms between 2000 and 2018 ranging from 3.6% to 6.4%, while the cancer incidence in the country was projected to rise by 12% from 2020 to 2025 [7, 12]. In the previous ICMR 2004 - cancer burden estimates for India, 5.9 million DALYs were arrived from NCRP-PBCR data and WHO-DisMod-II tool [8]. The current study estimates for 2016 are 22.6 million DALYs_AMI_, indicating an evident rise of more than 3 times cancer DALYs since 2004, primarily due to the growing and ageing population. Further, our study projections reveal a 11.4% rise in cancer DALYs from 2021 to 2025. However, these projections are influenced by the future investment decisions in health care, cancer research, public awareness on cancer risk factor reduction, other social, economic changes and cancer notifiability [26–28].

The cancer burden varies between regions within the country. The current study reports a high cancer burden in the northern region followed by the north-east. Breast cancer in females, lung cancer and oesophageal cancer in males contributed to the highest burden in northern and northeast region, respectively. The estimated DALYs (reported and adjusted MI) in the current study varied with those reported by GBD in 2016 [3, 29]. The resulting estimates are highly dependent on the data quality, sources of data, completeness of data (incidence and mortality), data collection period, methodology, statistical modelling and assumptions [3, 5, 30, 31]. Nevertheless, the reliability on the NCRP is deemed to provide robust data to estimate national, regional and state burden on cancer as it adheres to standard methods established by the WHO-International Agency for Research in Cancer for the last 40 years [3, 6, 20]. NCRP has been the primary data source even for the GBD and the GLOBOCAN publications [3, 26]. With every 1% increase in adjusted MI from reported MI ratio, the DALYs per 100,000 increased by 37 for both sex, 36 for males and 39 for females. Furthermore, the inconsistencies between studies are not surprising even for high-income countries with complete cancer incidence and mortality data and, such variations in epidemiological analysis are common [31–34]. It is not possible to evaluate the causes for these inconsistencies as the complex constructed statistical models, as well as data adjustments used by GBD or other relevant studies, are not fully available in the public domain [31, 34].

The nations within a nation description of India best describes the heterogeneities in the epidemiological transition levels within its states. Most of the increase in cancer incidence in India can be attributed to its epidemiologic transition and the commitment of Government of India in improving the use of cancer diagnostics in the country. However, the burden imposed by cancer in India will continue to increase as a result of the continuing pace of cancer risk factors and its determinants. Maximum increases will occur in the most populous and least affluent states, where the current cancer diagnostic and treatment facilities are inadequate [22, 26, 35, 36].

In the current study, the YLLs contribution to DALYs is around 93% for all sites of cancer, and we have relied completely on the population-based registries for all cancer-specific data. Adding on to the merits of the current study is the use of recent 2012–16 period PBCR data to arrive at cancer burden metrics, that has not yet been utilized by any other study.

The study results were derived from the use of simple, locally relevant and reproducible methods replicable at the sub-national level for policy and planning purposes. However, the estimates are very sensitive to changes in the environment influenced by health insurance systems, certification of cause of death, number of cancer treating hospitals, number of cancers registrations and PBCR coverage. Despite the caveats, our results are the best available real-time and ongoing estimates of the cancer burden in India. They are best suited for use for priority setting and planning of cancer resource allocation and management across the nation [26].

Recognizing these inadequacies, focus on expansion and strengthening of available data systems in coverage, completeness, quality and access is an important investment to future research and development. Cross talk between data sources — mortality databases, cancer registration systems, Ayushman Bharat, Hospital Information System recording, and national as well as state cancer control programmes would play a significant role in strengthening the completeness of local data. Bringing in place cancer notifiability within the country will further strengthen the already available data through wider coverage with limited resource and funding [3, 6, 7].

## Conclusion

The detailed epidemiology of all cancer types by sex, age and at sub-national level described in this paper will be a blueprint for specifically targeted policy and program planning appropriate to the regional burden and needs. Concerted and sustained efforts for strategic allocation of resources in terms of finance and health infrastructure should be made for improved access to care, prevention, early detection and, management to meet the rising burden of cancer in India. Also, quantifying burden through locally relevant, regionalized approaches helps make relevant decisions assess set targets of Sustainable Development Goals, Universal Health Coverage and National Multisectoral Action Plan for Prevention and Control of Common Noncommunicable Diseases. This study would also encourage other low-middle income countries to adopt similar methods for strengthening their cancer disease burden estimation needed for policy and program interventions.

## Supplementary Information

**Additional file 1.**

**Additional file 2.**

**Additional file 3.**

**Additional file 4.**

## Data Availability

Data is available within this paper and its supporting information files. The corresponding author can be contacted at ncdir@ncdirindia.org for further clarification if required.

## Abbreviations

NCRP: National Cancer Registry Program
PBCR: Population Based Cancer Registry
SRS: Sample Registration Systems
WHO: World Health Organization
YLLs: Years of Life Lost
YLDs: Years Lived with Disability
DALYs: Disability Adjusted Life Years
GBD: Global Burden of Disease
ICMR: Indian Council of Medical Research
NCDIR: National Centre for Disease Informatics and Research
NCD: Noncommunicable Disease
ASR: Age Standardized Rate
RMI: Reported Mortality-Incidence ratio
AMI: Adjusted Mortality-Incidence ratio
