# Supplementary material for: Burden of cancers in India - estimates of cancer crude incidence, YLLs, YLDs and DALYs for 2021 and 2025 based on National Cancer Registry Program
Source: BMC Cancer. 2022 May 11;22:527. doi: 10.1186/s12885-022-09578-1 (PMC9092762; doi:10.1186/s12885-022-09578-1)
Supplement: Supplementary file 1 — Additional file 1. [file 12885_2022_9578_MOESM1_ESM.docx]

**E. ADDITIONAL MATERIALS**

- Title of data: Additional Table1: Estimated age and sex-wise distribution of population at all 28 PBCRs for 2012-16.
- File format: DOC file
- Description of data: Estimated age and sex-wise distribution of population at all 28 PBCRs for 2012-16.
- Title of data: Additional Table 2: Life expectancy by age group and sex as per WHO.
- File format: DOC file
- Description of data: Life expectancy by age group and sex as per WHO.
- Title of data: Additional Table3: Site-specific burden of cancer (Incidence, Mortality) per 100,000 by sex.
- File format: DOC file
- Description of data: Site-specific burden of cancer (Incidence, Mortality) per 100,000 by sex.
- Title of data:
  - Additional Table 4a: Distribution of age-standardized cancer burden (YLLs, YLDs and DALYs) per 100,000 in India for both sexes.
  - Additional Table 4b: Distribution of age-standardized cancer burden (YLLs, YLDs and DALYs) per 100,000 in males.
  - Additional Table 4c: Distribution of age-standardized cancer burden (YLLs, YLDs and DALYs) per 100,000 in females.
- File format: DOC file
- Description of data:
  - Distribution of age-standardized cancer burden (YLLs, YLDs and DALYs) per 100,000 in India for both sexes.
  - Distribution of age-standardized cancer burden (YLLs, YLDs and DALYs) per 100,000 in males.
  - Distribution of age-standardized cancer burden (YLLs, YLDs and DALYs) per 100,000 in females.
- Title of data: Additional Table 5: Centre specific burden of cancer (Incidence, Mortality, YLLs, YLDs, DALYs) per 100,000 by sex.
- File format: DOC file
- Description of data: Centre specific burden of cancer (Incidence, Mortality, YLLs, YLDs, DALYs) per 100,000 by sex.
- Title of data: Additional Table 6: Site-specific burden of cancer (YLLs, YLDs, DALYs) per 100,000 by sex.
- File format: DOC file
- Description of data: Site-specific burden of cancer (YLLs, YLDs, DALYs) per 100,000 by sex.
- Title of data:
  - Additional Figure 1a: Summary of the steps in the national cancer burden estimation for India.
  - Additional Figure 1b: Distribution fitting for MI ratio (i) Males (ii) Females (iii) Goodness-of-fit criteria.
- File format: JPEG file
- Description of data:
  - Summary of the steps in the national cancer burden estimation for India.
  - Distribution fitting for MI ratio(i) Males (ii) Females (iii) Goodness-of-fit criteria.
- Title of data: Additional Figure 2: Total cancer burden (a) YLLs, (b) YLDs, (c) DALYs by age group and sex (%).
- File format: JPEG file
- Description of data: Total cancer burden(a) YLLs, (b) YLDs, (c) DALYs by age group and sex (%).
- Title of data: Additional Figure 3: Total cancer DALYs (%) by age group and site.
- File format: JPEG file
- Description of data: Total cancer DALYs (%) by age group and site.

**Additional Table 1: Estimated age and sex-wise distribution of population at all 28 PBCRs for 2012-16.**

| Age group | Population | | |
| --- | --- | --- | --- |
|  | **Males** | **Females** | **Total** |
| 0-4 | 20493059 | 18720644 | 39213703 |
| 5-9 | 21474250 | 19272096 | 40746346 |
| 10-14 | 22923645 | 20519530 | 43443175 |
| 15-19 | 24102248 | 21369879 | 45472127 |
| 20-24 | 26846864 | 25140480 | 51987344 |
| 25-29 | 26263436 | 25088754 | 51352190 |
| 30-34 | 22755139 | 21244445 | 43999584 |
| 35-39 | 20681122 | 19647303 | 40328425 |
| 40-44 | 17994259 | 16798654 | 34792913 |
| 45-49 | 15645954 | 14669153 | 30315107 |
| 50-54 | 12727760 | 11587717 | 24315477 |
| 55-59 | 10085573 | 9337927 | 19423500 |
| 60-64 | 8370702 | 8238751 | 16609453 |
| 65-69 | 5557228 | 5521566 | 11078794 |
| 70-74 | 3933326 | 3975354 | 7908680 |
| 75-79 | 2178028 | 2341554 | 4519582 |
| 80-84 | 1292018 | 1517685 | 2809703 |
| 85+ | 1130677 | 1360195 | 2490872 |
| Total | **264455288** | **246351687** | **510806975** |

**Additional Table 2: Life expectancy by age group and sex as per WHO**

| Age group | Males | Females |
| --- | --- | --- |
| 0-4 | 90.0 | 90.0 |
| 5-9 | 85.0 | 85.0 |
| 10-14 | 80.0 | 80.0 |
| 15-19 | 75.0 | 75.0 |
| 20-24 | 70.0 | 70.0 |
| 25-29 | 65.0 | 65.0 |
| 30-34 | 60.0 | 60.0 |
| 35-39 | 55.0 | 55.0 |
| 40-44 | 50.0 | 50.0 |
| 45-49 | 45.0 | 46.0 |
| 50-54 | 40.0 | 40.0 |
| 55-59 | 35.0 | 36.0 |
| 60-64 | 30.0 | 31.0 |
| 65-69 | 26.0 | 26.0 |
| 70-74 | 20.0 | 21.0 |
| 75-79 | 16.0 | 16.0 |
| 80-84 | 11.0 | 11.0 |
| 85+ | 5.0 | 5.0 |

**Additional Table 3: Site-specific burden of cancer (Incidence, Mortality) per 100,000 by sex**

| ICD-10 | Cancer site | Incidence | | | | Mortality | | | |
| --- | --- | --- | --- | --- | --- | --- | --- | --- | --- |
|  |  | **CR** | | **ASR** | | **CR** | | **ASR** | |
|  |  | **Males** | **Females** | **Males** | **Females** | **Males** | **Females** | **Males** | **Females** |
| C00 | Lip | 0.4 | 0.2 | 0.5 | 0.2 | 0.1 | 0.1 | 0.1 | 0.1 |
| C01-C02 | Tongue | 5.7 | 2.1 | 6.7 | 2.4 | 1.5 | 0.6 | 1.8 | 0.7 |
| C03-C06 | Mouth | 8.1 | 3.3 | 9.4 | 3.9 | 2.0 | 1.0 | 2.4 | 1.2 |
| C07-C08 | Salivary Gland | 0.5 | 0.4 | 0.6 | 0.4 | 0.1 | 0.1 | 0.1 | 0.1 |
| C09 | Tonsil | 1.1 | 0.2 | 1.3 | 0.3 | 0.3 | 0.1 | 0.4 | 0.1 |
| C10 | Other Oropharynx | 0.9 | 0.2 | 1.2 | 0.2 | 0.3 | 0.1 | 0.4 | 0.1 |
| C11 | Nasopharynx | 0.7 | 0.3 | 0.8 | 0.4 | 0.2 | 0.1 | 0.2 | 0.1 |
| C12-C13 | Hypopharynx | 2.6 | 0.7 | 3.4 | 0.8 | 0.8 | 0.2 | 1.1 | 0.2 |
| C14 | Pharynx Unspecified | 0.5 | 0.2 | 0.6 | 0.2 | 0.3 | 0.1 | 0.4 | 0.1 |
| C15 | Oesophagus | 5.5 | 3.3 | 7.0 | 4.0 | 2.3 | 1.3 | 3.0 | 1.6 |
| C16 | Stomach | 4.6 | 2.7 | 6.0 | 3.2 | 2.0 | 1.1 | 2.6 | 1.4 |
| C17 | Small Intestine | 0.3 | 0.2 | 0.4 | 0.3 | 0.1 | 0.1 | 0.1 | 0.1 |
| C18 | Colon | 2.8 | 2.2 | 3.6 | 2.7 | 0.9 | 0.7 | 1.2 | 0.9 |
| C19-C20 | Rectum | 2.8 | 2.0 | 3.5 | 2.4 | 0.8 | 0.6 | 1.0 | 0.7 |
| C21 | Anus and Anal cavity | 0.4 | 0.3 | 0.5 | 0.3 | 0.1 | 0.1 | 0.1 | 0.1 |
| C22 | Liver | 3.6 | 1.7 | 4.7 | 2.0 | 2.0 | 0.9 | 2.6 | 1.1 |
| C23-C24 | Gallbladder | 2.1 | 4.0 | 2.7 | 4.7 | 0.8 | 1.4 | 1.0 | 1.7 |
| C25 | Pancreas | 1.6 | 1.2 | 2.1 | 1.4 | 0.8 | 0.6 | 1.1 | 0.8 |
| C30-C31 | Nose, Sinuses | 0.4 | 0.2 | 0.5 | 0.3 | 0.1 | 0.1 | 0.1 | 0.1 |
| C32 | Larynx | 3.9 | 0.6 | 5.1 | 0.7 | 1.1 | 0.2 | 1.5 | 0.2 |
| C33-C34 | Lung | 10.2 | 4.3 | 13.6 | 5.2 | 4.9 | 2.0 | 6.6 | 2.5 |
| C37-C38 | Other Thoracic Organs | 0.2 | 0.2 | 0.3 | 0.2 | 0.1 | 0.1 | 0.1 | 0.1 |
| C40-C41 | Bone | 1.2 | 0.9 | 1.2 | 0.9 | 0.3 | 0.2 | 0.3 | 0.2 |
| C43 | Melanoma of Skin | 0.4 | 0.3 | 0.5 | 0.4 | 0.1 | 0.1 | 0.1 | 0.1 |
| C44 | Other Skin | 1.2 | 1.0 | 1.5 | 1.2 | 0.2 | 0.2 | 0.3 | 0.2 |
| C45 | Mesothelioma | 0.01 | 0.01 | 0.02 | 0.01 | 0.01 | 0.003 | 0.01 | 0.004 |
| C46 | Kaposi Sarcoma | 0.004 | 0.004 | 0.01 | 0.004 | 0.004 | 0.005 | 0.005 | 0.005 |
| C47+C49 | Connective & Soft Tissue | 1.1 | 1.0 | 1.2 | 1.0 | 0.2 | 0.2 | 0.2 | 0.2 |
| C50 | Breast | 0.8 | 28.4 | 0.9 | 31.9 | 0.2 | 5.3 | 0.2 | 6.1 |
| C51 | Vulva |  | 0.4 |  | 0.4 |  | 0.1 |  | 0.1 |
| C52 | Vagina |  | 0.4 |  | 0.5 |  | 0.1 |  | 0.1 |
| C53 | Cervix Uteri |  | 10.7 |  | 12.1 |  | 2.3 |  | 2.7 |
| C54 | Corpus Uteri |  | 3.6 |  | 4.2 |  | 0.4 |  | 0.5 |
| C55 | Uterus Unspecified |  | 0.6 |  | 0.7 |  | 0.2 |  | 0.3 |
| C56 | Ovary |  | 6.4 |  | 7.2 |  | 1.9 |  | 2.2 |
| C57 | Other Female Genital organs |  | 0.1 |  | 0.1 |  | 0.02 |  | 0.02 |
| C58 | Placenta |  | 0.1 |  | 0.1 |  | 0.01 |  | 0.01 |
| C60 | Penis | 0.8 |  | 0.9 |  | 0.2 |  | 0.2 |  |
| C61 | Prostate | 5.4 |  | 8.1 |  | 1.4 |  | 2.2 |  |
| C62 | Testis | 0.6 |  | 0.6 |  | 0.1 |  | 0.1 |  |
| C63 | Other Male Genital organs | 0.1 |  | 0.1 |  | 0.01 |  | 0.02 |  |
| C64 | Kidney | 1.6 | 0.8 | 2.0 | 0.9 | 0.4 | 0.2 | 0.6 | 0.2 |
| C65 | Renal Pelvis | 0.01 | 0.01 | 0.02 | 0.01 | 0.01 | 0.002 | 0.01 | 0.003 |
| C66 | Ureter | 0.02 | 0.02 | 0.03 | 0.02 | 0.01 | 0.003 | 0.01 | 0.004 |
| C67 | Bladder | 2.8 | 0.8 | 3.8 | 0.9 | 0.7 | 0.2 | 0.9 | 0.2 |
| C68 | Unspecified Urinary Organs | 0.04 | 0.02 | 0.1 | 0.02 | 0.02 | 0.01 | 0.03 | 0.01 |
| C69 | Eye | 0.2 | 0.1 | 0.2 | 0.2 | 0.02 | 0.01 | 0.03 | 0.01 |
| C70-C72 | Brain, Nervous System | 2.7 | 1.8 | 3.0 | 2.0 | 0.8 | 0.6 | 1.0 | 0.7 |
| C73 | Thyroid | 1.1 | 3.3 | 1.2 | 3.4 | 0.1 | 0.3 | 0.2 | 0.3 |
| C74 | Adrenal Gland | 0.1 | 0.1 | 0.1 | 0.1 | 0.02 | 0.02 | 0.03 | 0.02 |
| C81 | Hodgkin’s Disease | 0.9 | 0.5 | 1.0 | 0.6 | 0.2 | 0.1 | 0.2 | 0.1 |
| C82-C85 | Non-Hodgkin’s Lymphoma | 3.3 | 2.3 | 4.0 | 2.6 | 1.0 | 0.6 | 1.2 | 0.8 |
| C88 | Malignant Immunoproliferative Diseases | 0.01 | 0.01 | 0.01 | 0.01 | 0.01 | 0.004 | 0.01 | 0.004 |
| C90 | Multiple Myeloma | 1.4 | 1.0 | 1.8 | 1.3 | 0.4 | 0.4 | 0.6 | 0.4 |
| C91 | Lymphoid Leukaemia | 1.8 | 1.0 | 2.0 | 1.1 | 0.5 | 0.4 | 0.6 | 0.4 |
| C92-C94 | Myeloid Leukaemia | 2.0 | 1.6 | 2.3 | 1.7 | 0.8 | 0.7 | 1.0 | 0.7 |
| C95 | Leukaemia Unspecified | 0.4 | 0.3 | 0.5 | 0.4 | 0.3 | 0.2 | 0.3 | 0.2 |
| C96 | CMD, O&U LHM | 0.1 | 0.04 | 0.1 | 0.04 | 0.03 | 0.02 | 0.04 | 0.02 |
| O&U | Other and Unspecified | 6.5 | 5.0 | 8.3 | 5.9 | 3.8 | 3.2 | 4.9 | 3.8 |
| All Sites |  | **95.9** | **103.2** | **119.9** | **118.0** | **33.5** | **29.1** | **43.5** | **34.7** |
| *Abbreviations: CR – Crude Rate; ASR – Age Standardized Rate; CMD - Chronic Myeloproliferative Disease; O&U LHM - Other and unspecified malignant neoplasms of lymphoid, hematopoietic and related tissue; O&U includes the Sites (ICD-10:C26, C39, C48, C75, C76, C77, C78, C79, C80, C97)* | | | | | | | | | |

**Additional Table 4a: Distribution of age-standardized cancer burden (YLLs, YLDs and DALYs) per 100,000 in India for both sexes**

| **Region** | **YLLs** | **YLLs_AMI_** | **YLDs** | **YLDs_AMI_** | **DALYs** | **DALYs_AMI_** | **28 states and 2 UTs** | **YLLs** | **YLLs_AMI_** | **YLDs** | **YLDs_AMI_** | **DALYs** | **DALYs_AMI_** | **Registry** | **YLLs** | **YLLs_AMI_** | **YLDs** | **YLDs_AMI_** | **DALYs** | **DALYs_AMI_** |
| --- | --- | --- | --- | --- | --- | --- | --- | --- | --- | --- | --- | --- | --- | --- | --- | --- | --- | --- | --- | --- |
| **Southern region** | 1241 | 2038 | 112 | 100 | 1353 | 2138 | Karnataka | 1245 | 2202 | 123 | 110 | 1368 | 2312 | Bangalore | 1245 | 2202 | 123 | 110 | 1368 | 2312 |
|  |  |  |  |  |  |  | Telangana | 519 | 1892 | 111 | 86 | 630 | 1979 | Hyderabad District | 519 | 1892 | 111 | 86 | 630 | 1979 |
|  |  |  |  |  |  |  | Kerala | 1676 | 2004 | 105 | 101 | 1780 | 2104 | Kollam District | 1729 | 1886 | 97 | 94 | 1826 | 1980 |
|  |  |  |  |  |  |  |  |  |  |  |  |  |  | Thiruvananthapuram District | 1622 | 2121 | 113 | 107 | 1735 | 2228 |
|  |  |  |  |  |  |  | Tamil Nadu | 1091 | 2090 | 116 | 101 | 1206 | 2191 | Chennai | 1091 | 2090 | 116 | 101 | 1206 | 2191 |
|  |  |  |  |  |  |  | Andhra Pradesh | 1241 | 2038 | 112 | 100 | 1353 | 2138 |  |  |  |  |  |  |  |
| **Western Region** | 1042 | 1468 | 68 | 62 | 1110 | 1530 | Maharashtra | 1068 | 1440 | 66 | 61 | 1134 | 1500 | Mumbai | 1959 | 1952 | 89 | 89 | 2048 | 2041 |
|  |  |  |  |  |  |  |  |  |  |  |  |  |  | Barshi Rural | 1313 | 1318 | 43 | 43 | 1355 | 1361 |
|  |  |  |  |  |  |  | Goa | 1042 | 1468 | 68 | 62 | 1110 | 1530 | Osmanabad & Beed (Barshi Expanded) | 371 | 809 | 42 | 35 | 413 | 844 |
|  |  |  |  |  |  |  |  |  |  |  |  |  |  | Pune | 1132 | 1396 | 73 | 71 | 1205 | 1467 |
|  |  |  |  |  |  |  | Rajasthan | 1042 | 1468 | 68 | 62 | 1110 | 1530 | Aurangabad | 378 | 1288 | 76 | 63 | 454 | 1351 |
|  |  |  |  |  |  |  | Gujarat | 855 | 1665 | 82 | 70 | 936 | 1735 | Wardha District | 764 | 1569 | 87 | 53 | 851 | 1622 |
|  |  |  |  |  |  |  |  |  |  |  |  |  |  | Nagpur | 1559 | 1745 | 53 | 72 | 1612 | 1817 |
|  |  |  |  |  |  |  |  |  |  |  |  |  |  | Ahmedabad Urban | 855 | 1665 | 82 | 70 | 936 | 1735 |
| **Northern Region** | 1015 | 2304 | 123 | 103 | 1137 | 2408 | Punjab | 1127 | 2071 | 106 | 94 | 1232 | 2165 | Patiala District | 1127 | 2071 | 106 | 94 | 1232 | 2165 |
|  |  |  |  |  |  |  | Jammu and Kashmir | 1015 | 2304 | 123 | 103 | 1137 | 2408 |  |  |  |  |  |  |  |
|  |  |  |  |  |  |  | Haryana | 1015 | 2304 | 123 | 103 | 1137 | 2408 |  |  |  |  |  |  |  |
|  |  |  |  |  |  |  | Himachal Pradesh | 1015 | 2304 | 123 | 103 | 1137 | 2408 | Delhi | 902 | 2538 | 140 | 113 | 1043 | 2651 |
|  |  |  |  |  |  |  | Uttarakhand | 1015 | 2304 | 123 | 103 | 1137 | 2408 |  |  |  |  |  |  |  |
|  |  |  |  |  |  |  | Delhi | 902 | 2538 | 140 | 113 | 1043 | 2651 |  |  |  |  |  |  |  |
| **Central Region** | 1261 | 1941 | 90 | 83 | 1351 | 2024 | Uttar Pradesh | 1261 | 1941 | 90 | 83 | 1351 | 2024 | Bhopal | 1261 | 1941 | 90 | 83 | 1351 | 2024 |
|  |  |  |  |  |  |  | Madhya Pradesh | 1261 | 1941 | 90 | 83 | 1351 | 2024 |  |  |  |  |  |  |  |
|  |  |  |  |  |  |  | Chhattisgarh | 1261 | 1941 | 90 | 83 | 1351 | 2024 |  |  |  |  |  |  |  |
| **Eastern Region** | 1100 | 1343 | 72 | 70 | 1171 | 1412 | Odisha | 1100 | 1343 | 72 | 70 | 1171 | 1412 | Kolkata | 1100 | 1343 | 72 | 70 | 1171 | 1412 |
|  |  |  |  |  |  |  | Bihar | 1100 | 1343 | 72 | 70 | 1171 | 1412 |  |  |  |  |  |  |  |
|  |  |  |  |  |  |  | West Bengal | 1100 | 1343 | 72 | 70 | 1171 | 1412 |  |  |  |  |  |  |  |
|  |  |  |  |  |  |  | Jharkhand | 1100 | 1343 | 72 | 70 | 1171 | 1412 |  |  |  |  |  |  |  |
| **North Eastern Region** | 1310 | 2074 | 118 | 103 | 1428 | 2177 | Assam | 1057 | 2313 | 142 | 107 | 1198 | 2419 | Dibrugarh District | 687 | 1537 | 82 | 70 | 769 | 1607 |
|  |  |  |  |  |  |  |  |  |  |  |  |  |  | Kamrup Urban District | 1701 | 3216 | 177 | 159 | 1878 | 3375 |
|  |  |  |  |  |  |  |  |  |  |  |  |  |  | Cachar District | 782 | 2185 | 166 | 91 | 948 | 2276 |
|  |  |  |  |  |  |  | Arunachal Pradesh | 877 | 2055 | 156 | 140 | 1033 | 2195 | West Arunachal (Naharlagun) | 868 | 1946 | 92 | 78 | 960 | 2024 |
|  |  |  |  |  |  |  |  |  |  |  |  |  |  | Pasighat | 886 | 2164 | 220 | 201 | 1107 | 2365 |
|  |  |  |  |  |  |  | Meghalaya | 1997 | 2503 | 110 | 106 | 2108 | 2609 | Meghalaya | 1997 | 2503 | 110 | 106 | 2108 | 2609 |
|  |  |  |  |  |  |  | Manipur | 592 | 1103 | 66 | 53 | 657 | 1156 | Manipur State | 592 | 1103 | 66 | 53 | 657 | 1156 |
|  |  |  |  |  |  |  | Mizoram | 3126 | 3271 | 154 | 153 | 3279 | 3424 | Mizoram State | 3126 | 3271 | 154 | 153 | 3279 | 3424 |
|  |  |  |  |  |  |  | Nagaland | 905 | 1999 | 101 | 89 | 1006 | 2088 | Nagaland | 905 | 1999 | 101 | 89 | 1006 | 2088 |
|  |  |  |  |  |  |  | Sikkim | 1499 | 1524 | 79 | 79 | 1578 | 1602 | Sikkim State | 1499 | 1524 | 79 | 79 | 1578 | 1602 |
|  |  |  |  |  |  |  | Tripura | 1362 | 1364 | 55 | 55 | 1417 | 1419 | Tripura State | 1362 | 1364 | 55 | 55 | 1417 | 1419 |
| **ALL INDIA** | **1183** | **1820** | **95** | **87** | **1277** | **1908** |  | | | | | | | | | | | | | |

**Additional Table 4b: Distribution of age-standardized cancer burden (YLLs, YLDs and DALYs) per 100,000 in males**

| **Region** | **YLLs** | **YLLs_AMI_** | **YLDs** | **YLDs_AMI_** | **DALYs** | **DALYs_AMI_** | **28 states and 2 UTs** | **YLLs** | **YLLs_AMI_** | **YLDs** | **YLDs_AMI_** | **DALYs** | **DALYs_AMI_** | **Registry** | **YLLs** | **YLLs_AMI_** | **YLDs** | **YLDs_AMI_** | **DALYs** | **DALYs_AMI_** |
| --- | --- | --- | --- | --- | --- | --- | --- | --- | --- | --- | --- | --- | --- | --- | --- | --- | --- | --- | --- | --- |
| **Southern region** | 1395 | 2003 | 104 | 97 | 1499 | 2100 | Karnataka | 1228 | 1978 | 107 | 99 | 1335 | 2078 | Bangalore | 1228 | 1978 | 107 | 99 | 1335 | 2078 |
|  |  |  |  |  |  |  | Telangana | 587 | 1690 | 92 | 76 | 679 | 1766 | Hyderabad District | 587 | 1690 | 92 | 76 | 679 | 1766 |
|  |  |  |  |  |  |  | Kerala | 1991 | 2175 | 107 | 106 | 2098 | 2281 | Kollam District | 2110 | 2110 | 102 | 102 | 2211 | 2211 |
|  |  |  |  |  |  |  |  |  |  |  |  |  |  | Thiruvananthapuram District | 1872 | 2241 | 112 | 111 | 1984 | 2351 |
|  |  |  |  |  |  |  | Tamil Nadu | 1177 | 1996 | 106 | 97 | 1283 | 2093 | Chennai | 1177 | 1996 | 106 | 97 | 1283 | 2093 |
|  |  |  |  |  |  |  | Andhra Pradesh | 1395 | 2003 | 104 | 97 | 1499 | 2100 |  |  |  |  |  |  |  |
| **Western Region** | 1075 | 1482 | 67 | 62 | 1141 | 1544 | Maharashtra | 1080 | 1424 | 63 | 59 | 1143 | 1483 | Mumbai | 1949 | 1939 | 87 | 87 | 2036 | 2026 |
|  |  |  |  |  |  |  |  |  |  |  |  |  |  | Barshi Rural | 1297 | 1298 | 40 | 40 | 1336 | 1337 |
|  |  |  |  |  |  |  | Goa | 1075 | 1482 | 67 | 62 | 1141 | 1544 | Osmanabad & Beed (Barshi Expanded) | 348 | 715 | 37 | 31 | 385 | 746 |
|  |  |  |  |  |  |  |  |  |  |  |  |  |  | Pune | 1076 | 1305 | 69 | 68 | 1144 | 1373 |
|  |  |  |  |  |  |  | Rajasthan | 1075 | 1482 | 67 | 62 | 1141 | 1544 | Aurangabad | 441 | 1288 | 73 | 66 | 515 | 1354 |
|  |  |  |  |  |  |  |  |  |  |  |  |  |  | Wardha District | 1624 | 1623 | 51 | 51 | 1675 | 1674 |
|  |  |  |  |  |  |  | Gujarat | 1036 | 1894 | 90 | 79 | 1126 | 1974 | Nagpur | 827 | 1797 | 86 | 73 | 913 | 1869 |
|  |  |  |  |  |  |  |  |  |  |  |  |  |  | Ahmedabad Urban | 1036 | 1894 | 90 | 79 | 1126 | 1974 |
| **Northern Region** | 1064 | 2287 | 119 | 102 | 1183 | 2389 | Punjab | 1147 | 1959 | 97 | 89 | 1244 | 2048 | Patiala District | 1147 | 1959 | 97 | 89 | 1244 | 2048 |
|  |  |  |  |  |  |  | Jammu and Kashmir | 1064 | 2287 | 119 | 102 | 1183 | 2389 |  |  |  |  |  |  |  |
|  |  |  |  |  |  |  | Haryana | 1064 | 2287 | 119 | 102 | 1183 | 2389 |  |  |  |  |  |  |  |
|  |  |  |  |  |  |  | Himachal Pradesh | 1064 | 2287 | 119 | 102 | 1183 | 2389 | Delhi | 981 | 2614 | 142 | 116 | 1123 | 2730 |
|  |  |  |  |  |  |  | Uttarakhand | 1064 | 2287 | 119 | 102 | 1183 | 2389 |  |  |  |  |  |  |  |
|  |  |  |  |  |  |  | Delhi | 981 | 2614 | 142 | 116 | 1123 | 2730 |  |  |  |  |  |  |  |
| **Central Region** | 1409 | 1920 | 86 | 83 | 1495 | 2003 | Uttar Pradesh | 1409 | 1920 | 86 | 83 | 1495 | 2003 | Bhopal | 1409 | 1920 | 86 | 83 | 1495 | 2003 |
|  |  |  |  |  |  |  | Madhya Pradesh | 1409 | 1920 | 86 | 83 | 1495 | 2003 |  |  |  |  |  |  |  |
|  |  |  |  |  |  |  | Chhattisgarh | 1409 | 1920 | 86 | 83 | 1495 | 2003 |  |  |  |  |  |  |  |
| **Eastern Region** | 1150 | 1335 | 72 | 71 | 1222 | 1406 | Odisha | 1150 | 1335 | 72 | 71 | 1222 | 1406 | Kolkata | 1150 | 1335 | 72 | 71 | 1222 | 1406 |
|  |  |  |  |  |  |  | Bihar | 1150 | 1335 | 72 | 71 | 1222 | 1406 |  |  |  |  |  |  |  |
|  |  |  |  |  |  |  | West Bengal | 1150 | 1335 | 72 | 71 | 1222 | 1406 |  |  |  |  |  |  |  |
|  |  |  |  |  |  |  | Jharkhand | 1150 | 1335 | 72 | 71 | 1222 | 1406 |  |  |  |  |  |  |  |
| **North Eastern Region** | 1554 | 2268 | 129 | 117 | 1683 | 2384 | Assam | 1276 | 2466 | 148 | 114 | 1424 | 2580 | Dibrugarh District | 835 | 1607 | 83 | 73 | 919 | 1680 |
|  |  |  |  |  |  |  |  |  |  |  |  |  |  | Kamrup Urban District | 2119 | 3483 | 187 | 174 | 2306 | 3657 |
|  |  |  |  |  |  |  |  |  |  |  |  |  |  | Cachar District | 875 | 2308 | 173 | 94 | 1048 | 2402 |
|  |  |  |  |  |  |  | Arunachal Pradesh | 984 | 2028 | 182 | 169 | 1166 | 2197 | West Arunachal (Naharlagun) | 1014 | 1943 | 90 | 80 | 1104 | 2024 |
|  |  |  |  |  |  |  |  |  |  |  |  |  |  | Pasighat | 954 | 2113 | 273 | 258 | 1228 | 2371 |
|  |  |  |  |  |  |  | Meghalaya | 2618 | 3386 | 141 | 138 | 2759 | 3524 | Meghalaya | 2618 | 3386 | 141 | 138 | 2759 | 3524 |
|  |  |  |  |  |  |  | Manipur | 616 | 1010 | 55 | 58 | 671 | 1069 | Manipur State | 616 | 1010 | 55 | 58 | 671 | 1069 |
|  |  |  |  |  |  |  | Mizoram | 3826 | 3836 | 166 | 166 | 3992 | 4002 | Mizoram State | 3826 | 3836 | 166 | 166 | 3992 | 4002 |
|  |  |  |  |  |  |  | Nagaland | 1239 | 2241 | 107 | 98 | 1346 | 2339 | Nagaland | 1239 | 2241 | 107 | 98 | 1346 | 2339 |
|  |  |  |  |  |  |  | Sikkim | 1422 | 1440 | 79 | 79 | 1501 | 1519 | Sikkim State | 1422 | 1440 | 79 | 79 | 1501 | 1519 |
|  |  |  |  |  |  |  | Tripura | 1576 | 1578 | 64 | 64 | 1640 | 1642 | Tripura State | 1576 | 1578 | 64 | 64 | 1640 | 1642 |
| **ALL INDIA** | **1269** | **1830** | **92** | **88** | **1360** | **1918** |  | | | | | | | | | | | | | |

**Additional Table 4c: Distribution of age-standardized cancer burden (YLLs, YLDs and DALYs) per 100,000 in females**

| **Region** | **YLLs** | **YLLs_AMI_** | **YLDs** | **YLDs_AMI_** | **DALYs** | **DALYs_AMI_** | **28 states and 2 UTs** | **YLLs** | **YLLs_AMI_** | **YLDs** | **YLDs_AMI_** | **DALYs** | **DALYs_AMI_** | **Registry** | **YLLs** | **YLLs_AMI_** | **YLDs** | **YLDs_AMI_** | **DALYs** | **DALYs_AMI_** |
| --- | --- | --- | --- | --- | --- | --- | --- | --- | --- | --- | --- | --- | --- | --- | --- | --- | --- | --- | --- | --- |
| **Southern region** | 1088 | 2093 | 120 | 104 | 1207 | 2196 | Karnataka | 1262 | 2449 | 139 | 121 | 1402 | 2570 | Bangalore | 1262 | 2449 | 139 | 121 | 1402 | 2570 |
|  |  |  |  |  |  |  | Telangana | 451 | 2114 | 129 | 98 | 580 | 2212 | Hyderabad District | 451 | 2114 | 129 | 98 | 580 | 2212 |
|  |  |  |  |  |  |  | Kerala | 1360 | 1856 | 102 | 96 | 1463 | 1952 | Kollam District | 1348 | 1695 | 92 | 88 | 1440 | 1784 |
|  |  |  |  |  |  |  |  |  |  |  |  |  |  | Thiruvananthapuram District | 1373 | 2017 | 113 | 105 | 1485 | 2121 |
|  |  |  |  |  |  |  | Tamil Nadu | 1004 | 2190 | 125 | 106 | 1129 | 2296 | Chennai | 1004 | 2190 | 125 | 106 | 1129 | 2296 |
|  |  |  |  |  |  |  | Andhra Pradesh | 1088 | 2093 | 120 | 104 | 1207 | 2196 |  |  |  |  |  |  |  |
| **Western Region** | 1008 | 1456 | 70 | 63 | 1078 | 1519 | Maharashtra | 1056 | 1461 | 69 | 63 | 1125 | 1524 | Mumbai | 1969 | 1978 | 92 | 92 | 2061 | 2070 |
|  |  |  |  |  |  |  |  |  |  |  |  |  |  | Barshi Rural | 1329 | 1331 | 46 | 46 | 1374 | 1377 |
|  |  |  |  |  |  |  | Goa | 1008 | 1456 | 70 | 63 | 1078 | 1519 | Osmanabad & Beed (Barshi Expanded) | 394 | 905 | 47 | 39 | 441 | 944 |
|  |  |  |  |  |  |  |  |  |  |  |  |  |  | Pune | 1187 | 1498 | 78 | 74 | 1265 | 1572 |
|  |  |  |  |  |  |  | Rajasthan | 1008 | 1456 | 70 | 63 | 1078 | 1519 | Aurangabad | 315 | 1302 | 79 | 62 | 393 | 1364 |
|  |  |  |  |  |  |  |  |  |  |  |  |  |  | Wardha District | 1499 | 1520 | 55 | 55 | 1554 | 1575 |
|  |  |  |  |  |  |  | Gujarat | 673 | 1421 | 73 | 61 | 746 | 1483 | Nagpur | 701 | 1696 | 88 | 72 | 789 | 1768 |
|  |  |  |  |  |  |  |  |  |  |  |  |  |  | Ahmedabad Urban | 673 | 1421 | 73 | 61 | 746 | 1483 |
| **Northern Region** | 965 | 2328 | 127 | 105 | 1092 | 2432 | Punjab | 1106 | 2189 | 115 | 99 | 1221 | 2288 | Patiala District | 1106 | 2189 | 115 | 99 | 1221 | 2288 |
|  |  |  |  |  |  |  | Jammu and Kashmir | 965 | 2328 | 127 | 105 | 1092 | 2432 |  |  |  |  |  |  |  |
|  |  |  |  |  |  |  | Haryana | 965 | 2328 | 127 | 105 | 1092 | 2432 |  |  |  |  |  |  |  |
|  |  |  |  |  |  |  | Himachal Pradesh | 965 | 2328 | 127 | 105 | 1092 | 2432 | Delhi | 824 | 2466 | 138 | 110 | 962 | 2576 |
|  |  |  |  |  |  |  | Uttarakhand | 965 | 2328 | 127 | 105 | 1092 | 2432 |  |  |  |  |  |  |  |
|  |  |  |  |  |  |  | Delhi | 824 | 2466 | 138 | 110 | 962 | 2576 |  |  |  |  |  |  |  |
| **Central Region** | 1112 | 1970 | 95 | 84 | 1207 | 2053 | Uttar Pradesh | 1112 | 1970 | 95 | 84 | 1207 | 2053 | Bhopal | 1112 | 1970 | 95 | 84 | 1207 | 2053 |
|  |  |  |  |  |  |  | Madhya Pradesh | 1112 | 1970 | 95 | 84 | 1207 | 2053 |  |  |  |  |  |  |  |
|  |  |  |  |  |  |  | Chhattisgarh | 1112 | 1970 | 95 | 84 | 1207 | 2053 |  |  |  |  |  |  |  |
| **Eastern Region** | 1049 | 1355 | 72 | 69 | 1121 | 1423 | Odisha | 1049 | 1355 | 72 | 69 | 1121 | 1423 | Kolkata | 1049 | 1355 | 72 | 69 | 1121 | 1423 |
|  |  |  |  |  |  |  | Bihar | 1049 | 1355 | 72 | 69 | 1121 | 1423 |  |  |  |  |  |  |  |
|  |  |  |  |  |  |  | West Bengal | 1049 | 1355 | 72 | 69 | 1121 | 1423 |  |  |  |  |  |  |  |
|  |  |  |  |  |  |  | Jharkhand | 1049 | 1355 | 72 | 69 | 1121 | 1423 |  |  |  |  |  |  |  |
| **North Eastern Region** | 1065 | 1879 | 108 | 89 | 1173 | 1968 | Assam | 837 | 2140 | 135 | 99 | 972 | 2238 | Dibrugarh District | 538 | 1463 | 81 | 67 | 620 | 1530 |
|  |  |  |  |  |  |  |  |  |  |  |  |  |  | Kamrup Urban District | 1284 | 2905 | 166 | 142 | 1450 | 3046 |
|  |  |  |  |  |  |  |  |  |  |  |  |  |  | Cachar District | 689 | 2051 | 158 | 87 | 847 | 2138 |
|  |  |  |  |  |  |  | Arunachal Pradesh | 770 | 2093 | 130 | 108 | 901 | 2201 | West Arunachal (Naharlagun) | 722 | 1954 | 93 | 76 | 816 | 2030 |
|  |  |  |  |  |  |  |  |  |  |  |  |  |  | Pasighat | 818 | 2232 | 167 | 140 | 986 | 2372 |
|  |  |  |  |  |  |  | Meghalaya | 1377 | 1685 | 79 | 77 | 1456 | 1763 | Meghalaya | 1377 | 1685 | 79 | 77 | 1456 | 1763 |
|  |  |  |  |  |  |  | Manipur | 567 | 1196 | 76 | 49 | 643 | 1244 | Manipur State | 567 | 1196 | 76 | 49 | 643 | 1244 |
|  |  |  |  |  |  |  | Mizoram | 2425 | 2698 | 142 | 141 | 2567 | 2839 | Mizoram State | 2425 | 2698 | 142 | 141 | 2567 | 2839 |
|  |  |  |  |  |  |  | Nagaland | 571 | 1713 | 94 | 78 | 665 | 1791 | Nagaland | 571 | 1713 | 94 | 78 | 665 | 1791 |
|  |  |  |  |  |  |  | Sikkim | 1576 | 1619 | 79 | 79 | 1655 | 1698 | Sikkim State | 1576 | 1619 | 79 | 79 | 1655 | 1698 |
|  |  |  |  |  |  |  | Tripura | 1149 | 1151 | 46 | 46 | 1195 | 1197 | Tripura State | 1149 | 1151 | 46 | 46 | 1195 | 1197 |
| **ALL INDIA** | **1097** | **1817** | **98** | **87** | **1196** | **1904** |  | | | | | | | | | | | | | |

**Additional Table 5: Centre specific burden of cancer (Incidence, Mortality, YLLs, YLDs, DALYs) per 100,000 by sex**

| Region | Incidence | | | | Mortality | | | | YLL | | | | YLD | | | | DALYs | | | |
| --- | --- | --- | --- | --- | --- | --- | --- | --- | --- | --- | --- | --- | --- | --- | --- | --- | --- | --- | --- | --- |
|  | **CR** | | **ASR** | | **CR** | | **ASR** | | **CR** | | **ASR** | | **CR** | | **ASR** | | **CR** | | **ASR** | |
|  | **Males** | **Females** | **Males** | **Females** | **Males** | **Females** | **Males** | **Females** | **Males** | **Females** | **Males** | **Females** | **Males** | **Females** | **Males** | **Females** | **Males** | **Females** | **Males** | **Females** |
| Bangalore | 97.6 | 129.2 | 135.9 | 164.6 | 30.3 | 30.9 | 43.8 | 41.7 | 951.4 | 1020.1 | 1228.3 | 1262.4 | 77.4 | 109.6 | 106.8 | 139.3 | 1028.9 | 1129.7 | 1335.1 | 1401.8 |
| Mumbai | 96.4 | 116.6 | 120.1 | 126.2 | 55.6 | 60.0 | 72.0 | 67.0 | 1669.2 | 1859.1 | 1949.2 | 1968.8 | 70.2 | 84.9 | 87.0 | 91.9 | 1739.4 | 1944.0 | 2036.2 | 2060.7 |
| Chennai | 121.8 | 141.4 | 132.6 | 144.3 | 36.3 | 30.5 | 40.4 | 32.1 | 1150.9 | 997.5 | 1177.0 | 1004.1 | 97.7 | 122.7 | 106.4 | 125.3 | 1248.6 | 1120.2 | 1283.4 | 1129.5 |
| Hyderabad District | 75.3 | 96.7 | 102.2 | 131.8 | 13.3 | 9.7 | 18.8 | 13.8 | 464.7 | 350.3 | 587.2 | 451.0 | 68.6 | 94.0 | 92.3 | 129.1 | 533.3 | 444.2 | 679.5 | 580.1 |
| Bhopal | 83.4 | 90.0 | 111.2 | 114.9 | 31.0 | 23.8 | 42.4 | 31.2 | 1127.2 | 909.2 | 1409.4 | 1112.0 | 64.9 | 74.4 | 85.5 | 95.1 | 1192.1 | 983.6 | 1495.0 | 1207.1 |
| Delhi | 112.6 | 118.4 | 160.0 | 149.7 | 21.3 | 18.5 | 30.8 | 24.3 | 792.4 | 701.6 | 981.1 | 823.8 | 99.4 | 108.4 | 141.9 | 138.3 | 891.8 | 810.0 | 1123.0 | 962.2 |
| Barshi Rural | 53.9 | 67.2 | 55.3 | 65.0 | 38.7 | 42.3 | 39.2 | 39.5 | 1246.9 | 1363.1 | 1296.8 | 1328.5 | 38.6 | 47.5 | 39.6 | 45.9 | 1285.5 | 1410.5 | 1336.4 | 1374.4 |
| Kollam District | 159.4 | 139.1 | 139.8 | 115.9 | 84.3 | 51.6 | 74.0 | 42.1 | 2461.3 | 1611.0 | 2109.7 | 1348.4 | 115.6 | 110.2 | 101.7 | 92.0 | 2576.9 | 1721.2 | 2211.4 | 1440.4 |
| Aurangabad | 56.6 | 62.9 | 77.4 | 80.8 | 9.7 | 7.1 | 15.7 | 9.8 | 319.3 | 255.3 | 441.5 | 314.7 | 55.6 | 60.9 | 73.4 | 78.8 | 374.9 | 316.1 | 514.9 | 393.5 |
| Nagpur | 89.0 | 93.1 | 99.0 | 96.9 | 20.8 | 18.1 | 23.3 | 19.4 | 798.3 | 693.6 | 827.2 | 700.8 | 76.7 | 84.1 | 85.7 | 88.3 | 875.0 | 777.7 | 912.9 | 789.1 |
| Pune | 67.5 | 83.3 | 93.5 | 102.6 | 28.2 | 30.8 | 40.8 | 39.5 | 838.0 | 999.0 | 1075.6 | 1187.4 | 50.0 | 63.1 | 68.7 | 77.6 | 888.0 | 1062.1 | 1144.3 | 1265.1 |
| Thiruvananthapuram District | 170.4 | 164.8 | 151.9 | 137.9 | 72.2 | 52.5 | 64.2 | 43.6 | 2156.4 | 1637.6 | 1872.1 | 1372.5 | 125.6 | 134.6 | 112.3 | 112.8 | 2282.1 | 1772.2 | 1984.4 | 1485.4 |
| Kolkata | 104.1 | 100.3 | 95.8 | 92.1 | 45.4 | 36.9 | 42.5 | 34.5 | 1268.0 | 1142.2 | 1150.2 | 1049.1 | 77.9 | 78.4 | 71.5 | 71.8 | 1346.0 | 1220.6 | 1221.7 | 1120.8 |
| Dibrugarh District | 72.5 | 66.0 | 101.1 | 82.8 | 19.1 | 11.7 | 26.9 | 15.5 | 673.0 | 449.4 | 835.4 | 538.1 | 58.7 | 66.2 | 83.1 | 81.4 | 731.8 | 515.6 | 918.5 | 619.6 |
| Kamrup Urban | 190.5 | 150.8 | 238.3 | 186.0 | 58.6 | 31.5 | 75.5 | 41.7 | 1873.6 | 1101.6 | 2118.9 | 1283.6 | 150.0 | 137.5 | 187.3 | 166.5 | 2023.6 | 1239.1 | 2306.2 | 1450.1 |
| Cachar District | 99.2 | 87.0 | 141.4 | 114.3 | 19.0 | 13.6 | 28.2 | 19.4 | 652.8 | 527.6 | 875.1 | 689.0 | 120.1 | 114.9 | 172.6 | 158.4 | 772.9 | 642.6 | 1047.7 | 847.4 |
| Manipur State | 47.0 | 57.8 | 70.1 | 77.7 | 14.7 | 12.9 | 23.4 | 19.4 | 424.7 | 414.6 | 616.4 | 566.6 | 37.8 | 61.2 | 55.1 | 76.2 | 462.5 | 475.8 | 671.5 | 642.8 |
| Mizoram State | 146.1 | 127.5 | 230.5 | 190.5 | 84.2 | 53.5 | 137.3 | 86.2 | 2609.7 | 1681.3 | 3826.0 | 2425.2 | 105.0 | 95.8 | 165.9 | 141.7 | 2714.7 | 1777.0 | 3991.9 | 2566.9 |
| Sikkim State | 69.9 | 75.3 | 100.6 | 107.4 | 35.9 | 34.2 | 52.9 | 51.9 | 1059.5 | 1159.1 | 1422.0 | 1575.8 | 57.9 | 55.9 | 78.9 | 79.0 | 1117.4 | 1215.0 | 1500.9 | 1654.8 |
| Ahmedabad Urban | 89.1 | 74.7 | 107.3 | 82.9 | 24.4 | 16.4 | 29.5 | 18.4 | 935.5 | 639.1 | 1036.2 | 672.8 | 74.2 | 65.4 | 90.1 | 73.0 | 1009.7 | 704.5 | 1126.3 | 745.8 |
| Wardha District | 70.4 | 78.7 | 69.9 | 75.0 | 46.4 | 41.7 | 46.2 | 40.0 | 1647.9 | 1551.3 | 1624.1 | 1498.8 | 51.2 | 57.2 | 50.9 | 54.7 | 1699.1 | 1608.5 | 1675.0 | 1553.5 |
| Tripura State | 67.0 | 52.0 | 88.7 | 62.8 | 37.6 | 25.4 | 51.0 | 31.4 | 1262.2 | 984.5 | 1575.7 | 1148.6 | 48.4 | 38.0 | 64.1 | 45.9 | 1310.6 | 1022.5 | 1639.8 | 1194.5 |
| Nagaland | 74.5 | 56.3 | 136.4 | 95.2 | 21.1 | 9.1 | 42.5 | 16.5 | 729.6 | 377.5 | 1239.2 | 570.8 | 58.8 | 59.1 | 106.9 | 94.2 | 788.4 | 436.5 | 1346.1 | 665.0 |
| Meghalaya | 92.6 | 55.7 | 192.5 | 105.8 | 36.5 | 21.6 | 78.5 | 42.5 | 1369.9 | 765.5 | 2617.9 | 1376.8 | 67.1 | 41.9 | 141.3 | 79.4 | 1437.0 | 807.4 | 2759.2 | 1456.1 |
| West Arunachal | 56.6 | 56.3 | 109.5 | 104.9 | 14.9 | 9.7 | 30.0 | 21.6 | 613.4 | 396.6 | 1013.7 | 722.2 | 45.9 | 50.3 | 89.9 | 93.3 | 659.3 | 446.9 | 1103.6 | 815.5 |
| Osmanabad & Beed | 38.6 | 52.4 | 42.3 | 52.3 | 9.5 | 10.7 | 10.2 | 10.4 | 321.1 | 386.8 | 348.2 | 394.3 | 33.3 | 46.5 | 36.6 | 46.6 | 354.4 | 433.3 | 384.8 | 440.9 |
| Pasighat | 90.7 | 88.1 | 131.8 | 125.9 | 20.9 | 15.1 | 34.0 | 24.3 | 644.1 | 577.6 | 954.4 | 818.3 | 236.5 | 123.5 | 273.4 | 167.3 | 880.6 | 701.1 | 1227.8 | 985.6 |
| Patiala District | 101.6 | 127.7 | 119.1 | 134.5 | 30.8 | 30.5 | 36.7 | 33.1 | 1015.7 | 1053.8 | 1146.9 | 1106.4 | 83.2 | 108.9 | 96.9 | 114.7 | 1098.9 | 1162.7 | 1243.7 | 1221.1 |
| All Registries | **95.9** | **103.2** | **119.9** | **118.0** | **33.5** | **29.1** | **43.5** | **34.7** | **1075.4** | **979.7** | **1268.5** | **1097.4** | **73.8** | **86.1** | **91.8** | **98.3** | **1149.1** | **1065.8** | **1360.4** | **1195.8** |
| *Abbreviations: CR – Crude Rate; ASR – Age-Standardized Rate* | | | | | | | | | | | | | | | | | | | | |

**Additional Table 6: Site-specific burden of cancer (YLLs, YLDs, DALYs) per 100,000 by sex**

| ICD-10 | Cancer site | YLLs - CR | | YLDs - CR | | DALYs - CR | |
| --- | --- | --- | --- | --- | --- | --- | --- |
|  |  | **Males** | **Females** | **Males** | **Females** | **Males** | **Females** |
| C00 | Lip | 3.1 | 1.4 | 0.4 | 0.2 | 3.4 | 1.6 |
| C01-C02 | Tongue | 55.3 | 18.4 | 4.9 | 1.7 | 60.3 | 20.1 |
| C03-C06 | Mouth | 73.4 | 29.6 | 6.9 | 2.9 | 80.3 | 32.5 |
| C07-C08 | Salivary Gland | 3.2 | 1.8 | 0.8 | 0.7 | 4.0 | 2.5 |
| C09 | Tonsil | 10.3 | 1.8 | 0.9 | 0.2 | 11.2 | 2.0 |
| C10 | Other Oropharynx | 10.2 | 1.8 | 0.8 | 0.2 | 11.0 | 2.0 |
| C11 | Nasopharynx | 7.1 | 3.1 | 0.6 | 0.3 | 7.7 | 3.5 |
| C12-C13 | Hypopharynx | 24.6 | 7.2 | 2.1 | 0.6 | 26.7 | 7.7 |
| C14 | Pharynx Unspecified | 9.6 | 3.1 | 0.4 | 0.2 | 10.0 | 3.2 |
| C15 | Oesophagus | 69.9 | 38.0 | 4.0 | 2.6 | 73.9 | 40.6 |
| C16 | Stomach | 59.9 | 37.3 | 3.5 | 2.0 | 63.4 | 39.3 |
| C17 | Small Intestine | 3.1 | 2.4 | 0.3 | 0.2 | 3.4 | 2.6 |
| C18 | Colon | 26.0 | 22.4 | 2.3 | 1.9 | 28.2 | 24.2 |
| C19-C20 | Rectum | 25.5 | 19.6 | 2.2 | 1.7 | 27.7 | 21.2 |
| C21 | Anus and Anal cavity | 3.4 | 2.6 | 0.4 | 0.3 | 3.8 | 2.9 |
| C22 | Liver | 59.0 | 28.3 | 2.6 | 1.2 | 61.6 | 29.5 |
| C23-C24 | Gallbladder | 24.6 | 48.4 | 1.7 | 3.1 | 26.3 | 51.5 |
| C25 | Pancreas | 23.9 | 17.6 | 1.2 | 0.9 | 25.2 | 18.5 |
| C30-C31 | Nose, Sinuses | 3.5 | 1.8 | 0.4 | 0.4 | 3.9 | 2.1 |
| C32 | Larynx | 31.3 | 4.8 | 3.3 | 0.5 | 34.6 | 5.3 |
| C33-C34 | Lung | 137.7 | 60.0 | 7.4 | 3.1 | 145.1 | 63.1 |
| C37-C38 | Other Thoracic Organs | 3.5 | 2.4 | 0.3 | 0.1 | 3.7 | 2.6 |
| C40-C41 | Bone | 13.5 | 9.7 | 1.1 | 0.8 | 14.6 | 10.5 |
| C43 | Melanoma of Skin | 2.0 | 1.5 | 0.4 | 0.4 | 2.4 | 1.9 |
| C44 | Other Skin | 7.9 | 6.4 | 1.1 | 1.2 | 9.0 | 7.5 |
| C45 | Mesothelioma | 0.1 | 0.1 | 0.1 | 0.0 | 0.2 | 0.2 |
| C46 | Kaposi Sarcoma | 0.1 | 0.3 | 0.0 | 0.2 | 0.2 | 0.5 |
| C47+C49 | Connective & Soft Tissue | 8.1 | 6.9 | 1.0 | 0.9 | 9.1 | 7.8 |
| C50 | Breast | 4.8 | 185.0 | 0.7 | 25.7 | 5.5 | 210.7 |
| C51 | Vulva |  | 2.0 |  | 0.4 |  | 2.3 |
| C52 | Vagina |  | 2.8 |  | 0.4 |  | 3.2 |
| C53 | Cervix Uteri |  | 78.8 |  | 9.8 |  | 88.6 |
| C54 | Corpus Uteri |  | 12.6 |  | 3.3 |  | 15.9 |
| C55 | Uterus Unspecified |  | 6.8 |  | 0.5 |  | 7.3 |
| C56 | Ovary |  | 65.9 |  | 5.3 |  | 71.2 |
| C57 | Other Female Genital organs |  | 0.6 |  | 0.1 |  | 0.7 |
| C58 | Placenta |  | 0.3 |  | 0.7 |  | 1.0 |
| C60 | Penis | 4.6 |  | 0.7 |  | 5.3 |  |
| C61 | Prostate | 27.8 |  | 4.4 |  | 32.2 |  |
| C62 | Testis | 4.8 |  | 0.6 |  | 5.4 |  |
| C63 | Other Male Genital organs | 0.3 |  | 0.2 |  | 0.5 |  |
| C64 | Kidney | 13.8 | 6.4 | 1.5 | 0.7 | 15.3 | 7.1 |
| C65 | Renal Pelvis | 0.2 | 0.1 | 0.1 | 0.2 | 0.2 | 0.2 |
| C66 | Ureter | 0.2 | 0.1 | 0.1 | 0.1 | 0.2 | 0.2 |
| C67 | Bladder | 16.2 | 4.9 | 2.6 | 0.8 | 18.8 | 5.7 |
| C68 | Unspecified Urinary Organs | 0.6 | 0.2 | 0.1 | 0.1 | 0.7 | 0.3 |
| C69 | Eye | 1.4 | 0.7 | 0.3 | 0.4 | 1.7 | 1.2 |
| C70-C72 | Brain, Nervous System | 35.0 | 24.1 | 2.0 | 1.4 | 37.0 | 25.5 |
| C73 | Thyroid | 4.2 | 8.2 | 4.0 | 3.6 | 8.2 | 11.8 |
| C74 | Adrenal Gland | 1.1 | 0.9 | 0.5 | 0.2 | 1.5 | 1.2 |
| C81 | Hodgkin’s Disease | 7.0 | 4.3 | 0.9 | 0.6 | 7.9 | 4.9 |
| C82-C85 | Non-Hodgkin’s Lymphoma | 34.6 | 21.5 | 2.4 | 1.7 | 37.0 | 23.1 |
| C88 | Malignant Immunoproliferative Diseases | 0.3 | 0.3 | 0.0 | 0.0 | 0.3 | 0.3 |
| C90 | Multiple Myeloma | 12.4 | 10.8 | 1.1 | 0.9 | 13.4 | 11.7 |
| C91 | Lymphoid Leukaemia. | 29.7 | 19.5 | 1.6 | 0.9 | 31.3 | 20.4 |
| C92-C94 | Myeloid Leukaemia | 37.3 | 30.6 | 1.6 | 1.2 | 38.9 | 31.8 |
| C95 | Leukaemia Unspecified | 14.2 | 10.4 | 0.3 | 0.4 | 14.6 | 10.8 |
| C96 | CMD, O&U LHM | 1.2 | 0.7 | 0.1 | 0.1 | 1.3 | 0.8 |
| O&U | Other and Unspecified | 119.5 | 99.8 | 4.7 | 3.6 | 124.2 | 103.4 |
| All Sites |  | **1075.4** | **979.7** | **73.8** | **86.1** | **1149.1** | **1065.8** |
| *Abbreviations: CR – Crude Rate; CMD - Chronic Myeloproliferative Disease; O&U LHM - Other and unspecified malignant neoplasms of lymphoid, hematopoietic and related tissue; O&U includes the Sites (ICD-10:C26, C39, C48, C75, C76, C77, C78, C79, C80, C97)* | | | | | | | |
