# Supplementary figures and images for: Burden of cancers in India - estimates of cancer crude incidence, YLLs, YLDs and DALYs for 2021 and 2025 based on National Cancer Registry Program
Source: BMC Cancer. 2022 May 11;22:527. doi: 10.1186/s12885-022-09578-1 (PMC9092762; doi:10.1186/s12885-022-09578-1)

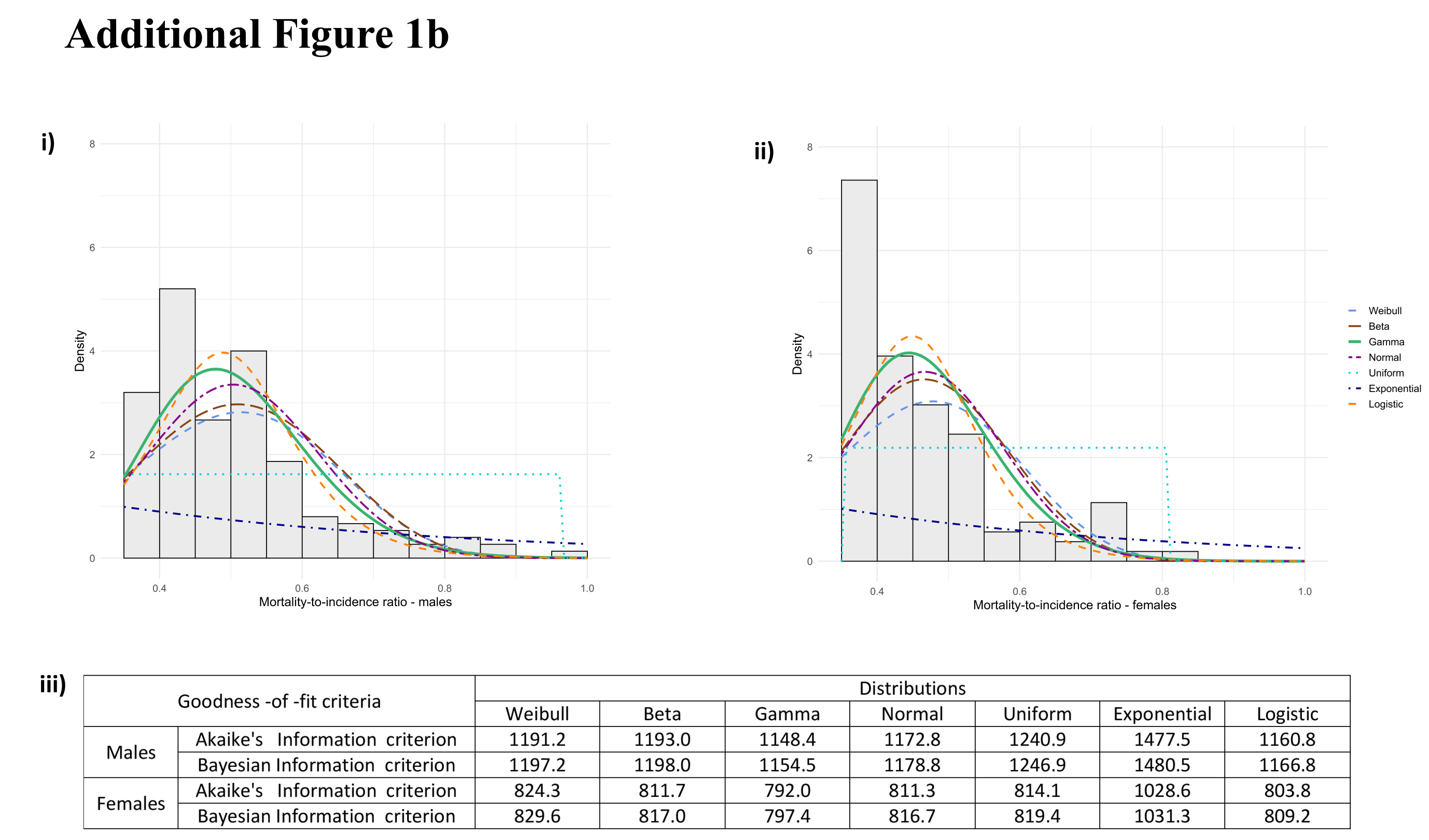

Supplement: Supplementary file 2 — Additional file 2. [file 12885_2022_9578_MOESM2_ESM.zip › Revised Additional Figure 1b_03_02_2022.tiff]

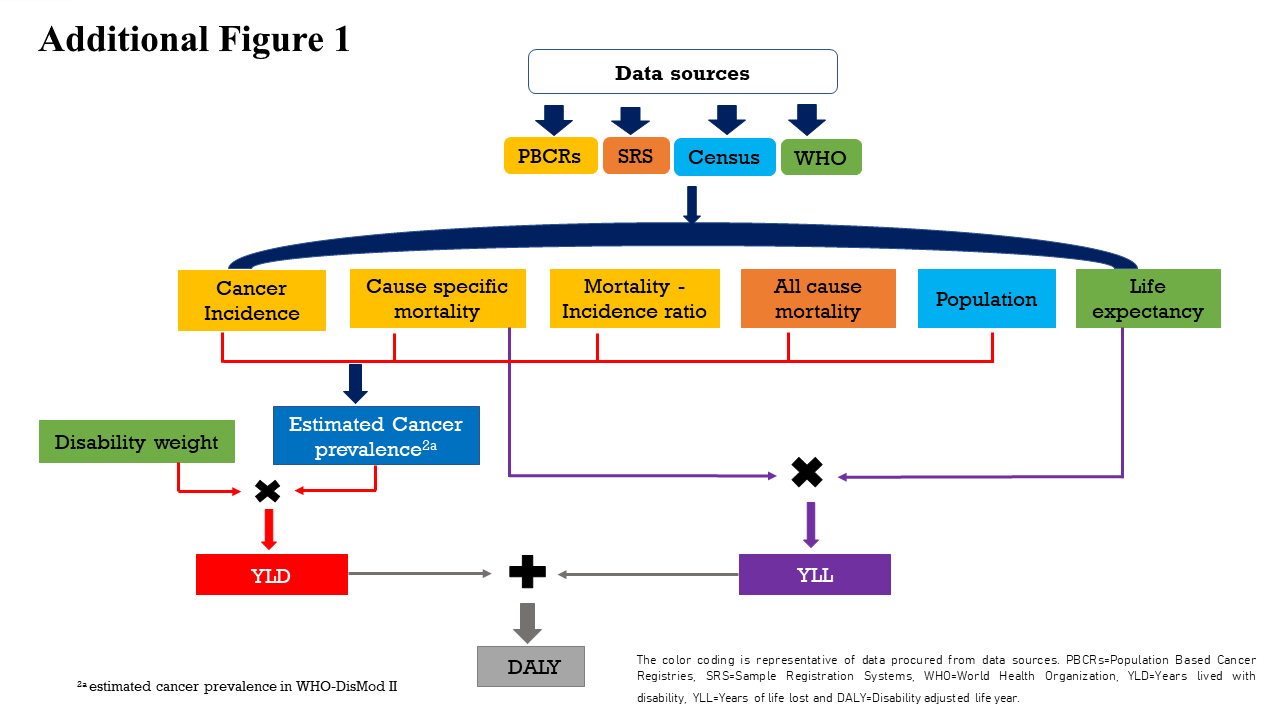

Supplement: Supplementary file 2 — Additional file 2. [file 12885_2022_9578_MOESM2_ESM.zip › Revised final additional fig_1_ESM.tiff]

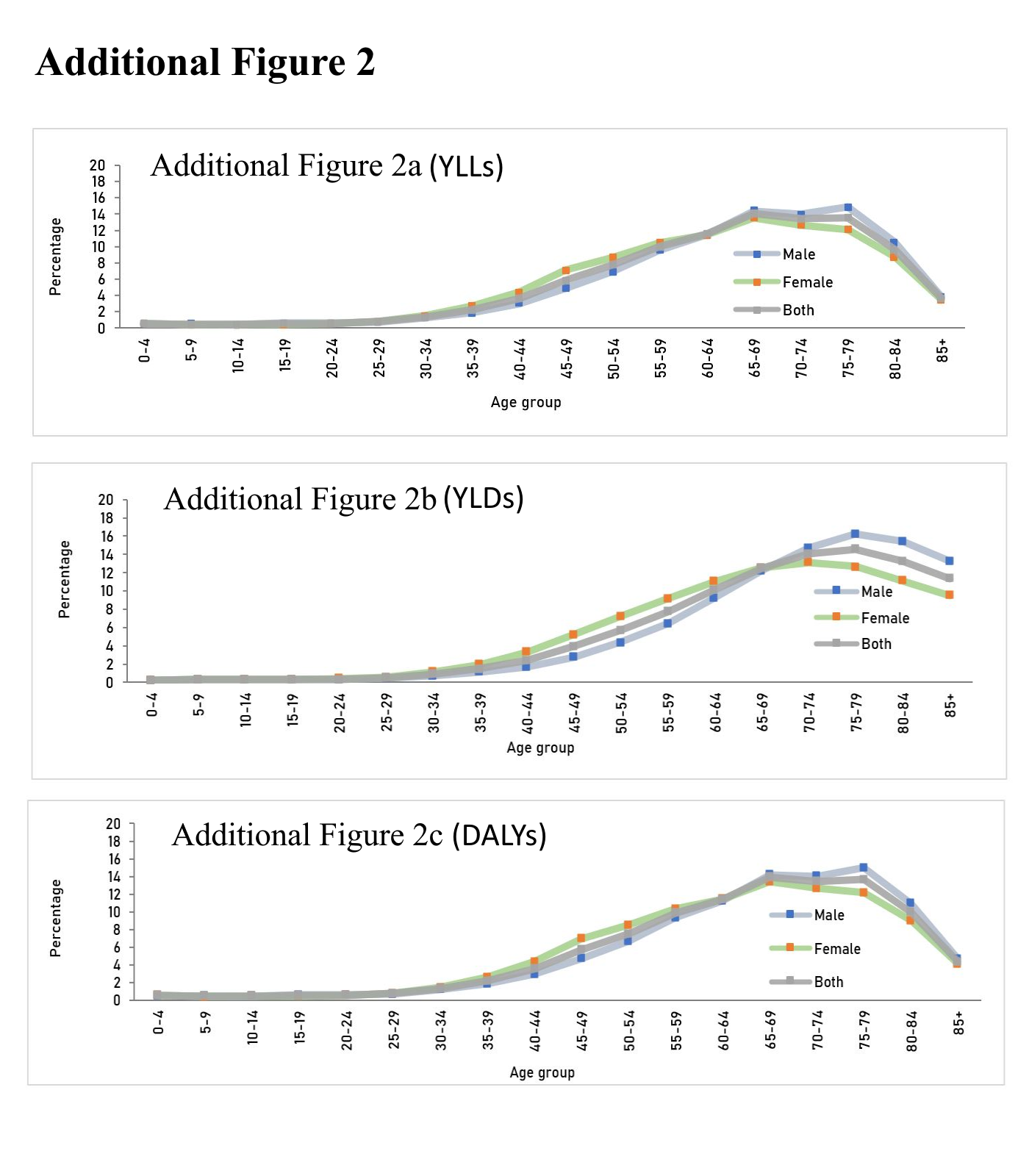

Supplement: Supplementary file 3 — Additional file 3. [file 12885_2022_9578_MOESM3_ESM.tiff]

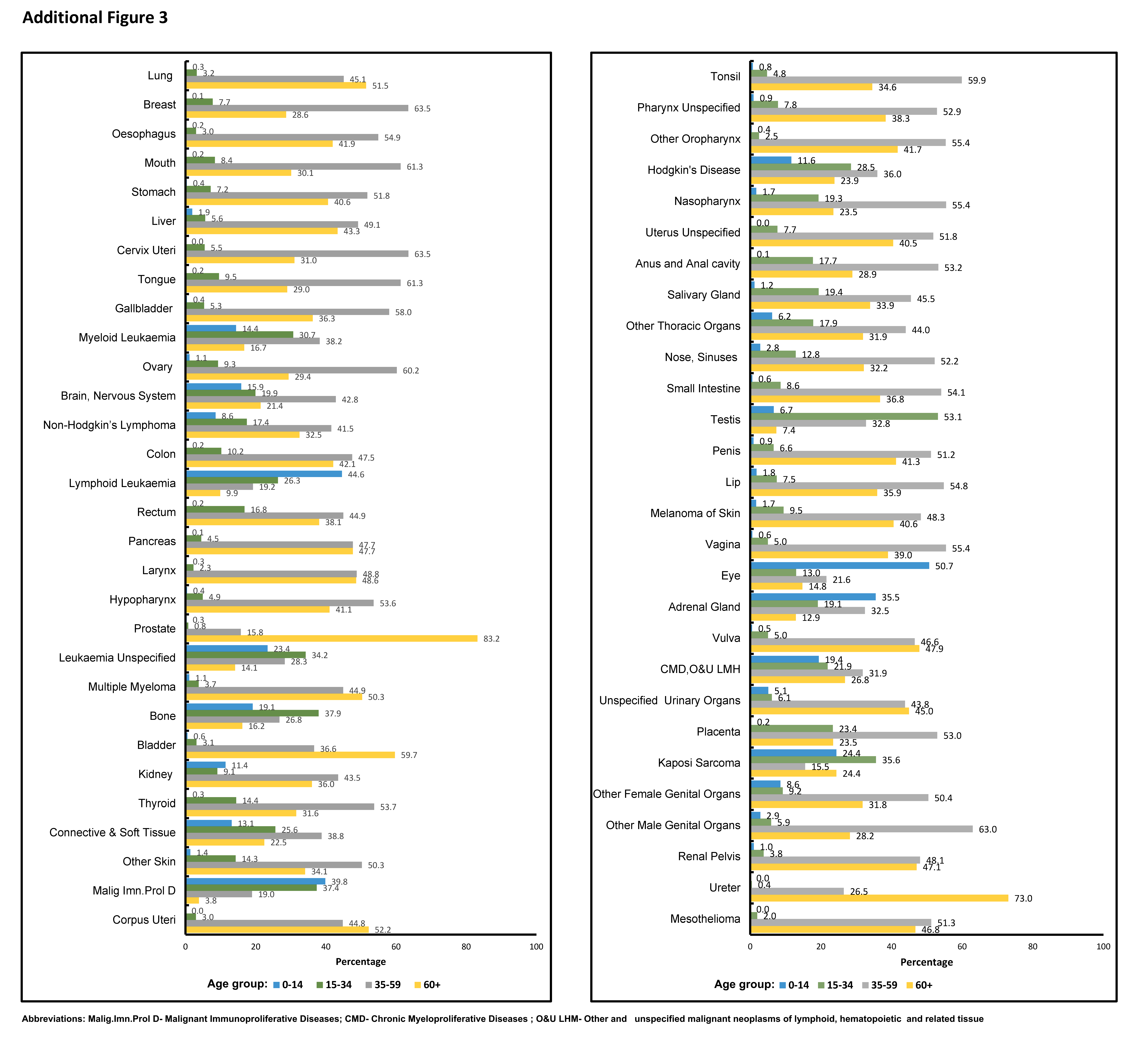

Supplement: Supplementary file 4 — Additional file 4. [file 12885_2022_9578_MOESM4_ESM.tiff]
